# The impact of Alzheimer’s disease risk factors on the pupillary light response

**DOI:** 10.3389/fnins.2023.1248640

**Published:** 2023-08-15

**Authors:** Sierra Sparks, Joana Pinto, Genevieve Hayes, Manuel Spitschan, Daniel P. Bulte

**Affiliations:** ^1^Department of Engineering Science, Institute of Biomedical Engineering, University of Oxford, Oxford, United Kingdom; ^2^TUM Department of Sport and Health Sciences (TUM SG), Chronobiology and Health, Technical University of Munich, Munich, Germany; ^3^TUM Institute for Advanced Study (TUM-IAS), Technical University of Munich, Garching, Germany; ^4^Max Planck Institute for Biological Cybernetics, Translational Sensory and Circadian Neuroscience, Tübingen, Germany

**Keywords:** Alzheimer’s disease, pupillary light response, dementia, neurodegeneration, risk factor, biomarkers, early detection, pupil

## Abstract

Alzheimer’s disease (AD) is the leading cause of dementia, and its prevalence is increasing and is expected to continue to increase over the next few decades. Because of this, there is an urgent requirement to determine a way to diagnose the disease, and to target interventions to delay and ideally stop the onset of symptoms, specifically those impacting cognition and daily livelihood. The pupillary light response (PLR) is controlled by the sympathetic and parasympathetic branches of the autonomic nervous system, and impairments to the pupillary light response (PLR) have been related to AD. However, most of these studies that assess the PLR occur in patients who have already been diagnosed with AD, rather than those who are at a higher risk for the disease but without a diagnosis. Determining whether the PLR is similarly impaired in subjects before an AD diagnosis is made and before cognitive symptoms of the disease begin, is an important step before using the PLR as a diagnostic tool. Specifically, identifying whether the PLR is impaired in specific at-risk groups, considering both genetic and non-genetic risk factors, is imperative. It is possible that the PLR may be impaired in association with some risk factors but not others, potentially indicating different pathways to neurodegeneration that could be distinguished using PLR. In this work, we review the most common genetic and lifestyle-based risk factors for AD and identify established relationships between these risk factors and the PLR. The evidence here shows that many AD risk factors, including traumatic brain injury, ocular and intracranial hypertension, alcohol consumption, depression, and diabetes, are directly related to changes in the PLR. Other risk factors currently lack sufficient literature to make any conclusions relating directly to the PLR but have shown links to impairments in the parasympathetic nervous system; further research should be conducted in these risk factors and their relation to the PLR.

## 1. Introduction

### 1.1. Alzheimer’s disease

With an ageing global population, the number of people living with dementia is increasing, and is projected to continue to increase over the next few decades, especially in those living in low- and middle-income countries (Livingston et al., 2017).

Importantly, Alzheimer’s disease (AD), which is the most common cause of dementia (Livingston et al., 2017), is believed to occur at least 20 years before symptoms of the disease arise (Association, 2019). It takes years of changes occurring in the brain before individuals with AD experience noticeable symptoms including memory loss and language problems (Association, 2019). As such, preventing Alzheimer’s depends primarily on understanding early steps in the disease’s pathogenesis, including an investigation into genetic factors and potential biomarkers that could be identified in its pre-symptomatic phase (Selkoe, 2012).

### 1.2. Alzheimer’s disease and the eye

Along with symptoms that affect cognition, patients who have been diagnosed with AD often display other biological characteristics, which can, to varying extents, be used in disease monitoring and potentially in diagnosis. One category of biological characteristics includes changes to the eye that occur in the early stages and during the progression of the disease, which will be explored further in this section.

#### 1.2.1. State of the art

Research into the eye and its relation to cognitive decline, including in both preclinical and onset AD, has been the focus of several recent studies and reviews. Many such reviews investigate changes and degeneration in the retina, and how this can relate to neurodegeneration (London et al., 2013; Cheung et al., 2017; den Haan et al., 2017; Alber et al., 2020; Ashok et al., 2020; Gupta et al., 2021; Snyder et al., 2021; Wang and Mao, 2021). These reviews discuss the role of amyloid-beta (Aβ), a biomarker for AD, in the retina and in patients with glaucoma (London et al., 2013; Ashok et al., 2020; Gupta et al., 2021; Snyder et al., 2021; Wang and Mao, 2021), and confounding factors in the eye such as retinal nerve fiber layer thickness thinning that can be observed in some cases of neurodegeneration (London et al., 2013; den Haan et al., 2017; Alber et al., 2020; Snyder et al., 2021). Other studies involving the retina also discuss accumulation of phosphorylated tau in the brain, another biomarker of AD, and how this often relates to an accumulation of tauopathy in the retina in cases of AD (London et al., 2013; Chiasseu et al., 2017; Ashok et al., 2020; Hart de Ruyter et al., 2023). In AD, tau-related changes cause retinal neuron dysfunction and subsequent death, which contributes to visual deficits in AD (Chiasseu et al., 2017). In cases of AD, tau related changes in the retina may be more consistent than amyloid beta changes in the retina, suggesting that phosphorylated tau in the retina may be a promising biomarker for AD (den Haan et al., 2018).

Despite its prevalence in current research, analyzing changes in the retina has established limitations as a diagnostic tool for AD and dementia because of the comorbidity with AD risk factors such as hypertension, diabetes, and retinopathy (Baker et al., 2007; Cheung et al., 2017; Bista Karki et al., 2020). As such, it can be difficult to differentiate damage done to the retina from these diseases from damage done due to potential neurodegeneration. Additionally, tests such as measuring the retinal nerve fiber thickness can lack sufficient specificity and sensitivity for broader clinical applications (Ashok et al., 2020).

Some studies have suggested that tracking eye movement abnormalities is an indicator of cognitive decline that can be used as a diagnostic tool for assessing the progression of AD (Boucart et al., 2014; Fernández et al., 2016). Others have analyzed the effects of tropicamide on the pupil dilation response, showing that the pupil dilation is altered in Alzheimer’s patients compared to healthy people (Robles et al., 1999); however, there have been other studies that have not shown this to be consistently statistically significant and so has limitations as a diagnostic aid (Kurz et al., 1997).

Further studies involving pupillometry applied to cognitive decline include identifying changes in the velocity and acceleration of pupil constriction in those with cognitive deficits (Stergiou et al., 2009). Pupillary changes have also been used to assess subject response to varying cognitive loads and the ability for those with and without cognitive impairment to adapt cognitive effort (Granholm et al., 2017).

#### 1.2.2. Pupillometry

The study of pupillometry has been around for many years. Granholm claims that changes in pupillary motility have been observed and used as indicators of medical state and emotional arousal for over two millennia (Granholm and Steinhauer, 2004). Loewenfeld cites Fontana’s work in 1765 as the earliest documentation of what was then known as “paradoxical pupil dilation”, or pupil dilation without changes in illumination (Loewenfeld, 1958). The work by Lowenstein and Loewenfeld was essential to the field of pupillometry, and their influential work is summarized in their textbook from 1999, The Pupil, which has been a standard reference on the pupil (Thompson, 2005).

Of note, the study of pupillometry has become increasingly more popular since the 1980s (Zandi et al., 2021). With this, there have been more studies that have looked at how various visual stimuli can be used to evoke a pupil response, and in turn what this response may be related to. For researchers in preventative medicine, pupillometry has been a valuable and inexpensive tool for screening for diseases such as diabetes and cardiac autonomic neuropathy (Lerner et al., 2015; Bista Karki et al., 2020).

The activity of human photoreceptors can control pupil size, which has best been shown by studies examining pupil size using the method of silent substitution - where pairs of lights are alternated to only stimulate one photoreceptor class at a time (Spitschan, 2019). These photoreceptors, consisting of melanopsin, rods, and cones, contribute to the control of the pupil in different ways, and in different temporal regimes (Spitschan, 2019). Between 1 and 10s from the onset of light exposure, cones and rods account for pupil constriction; melanopsin largely controls pupil size at 100 s, with some contribution from the rods (McDougal and Gamlin, 2010; Spitschan, 2019). Further, rods are not expected to contribute to pupil control at photopic light levels due to rod saturation (Aguilar and Stiles, 1954; Spitschan, 2019), while cone receptors and melanopsin-containing intrinsically photosensitive retinal ganglion cells (ipRGCs) are active during daylight and contribute to the constriction of the pupil (Spitschan et al., 2014).

When assessing potential pupillary changes in neurodegeneration, it is important to consider how these various psychological and physiological aspects may impact a response, and to account for these in a potential protocol.

##### 1.2.2.1. Pupillary light response

Outside of research assessing the retina’s role in neurodegeneration and general eye-tracking, there are other potential biomarkers involving the eyes that are important in AD research. Pupillometry at large has been applied extensively in the study of cognition (Eckstein et al., 2017).

Of particular interest to this review are the studies that have investigated changes in the pupillary light response (PLR) in subjects with cognitive impairment, which assesses how the pupil dilates and constricts in different light conditions (Lerner et al., 2015; Bista Karki et al., 2020). Although some studies have failed to show a relationship between an impaired PLR and cognitive impairment (Stergiou et al., 2009), there have been several studies that have suggested that changes to the PLR may occur in patients with AD (Fotiou et al., 2000), and even in patients in the preclinical phase of AD (Frost et al., 2017). These studies also suggest that dynamic pupillometry, and assessment of the PLR, could be useful tools in medical research to monitor the progression of cognitive decline, in addition to being used as a non-invasive, cost-effective screening tool for AD (Fotiou et al., 2000, 2008; Frost et al., 2017).

Most of the studies that assess the PLR occur in patients who have already been diagnosed with AD, rather than those who are at a higher risk for the disease due to genetic or non-genetic risk factors but who are not currently diagnosed with the disease. Because of this, it is difficult to determine whether the PLR is impaired because of the disease, or if an impaired PLR can indicate an elevated risk for later development of the disease. It would, therefore, be useful to know if the PLR is similarly impaired in subjects before an AD diagnosis is made and before cognitive symptoms of the disease begin. Specifically, it would be helpful to know whether the PLR is impaired in specific at-risk groups, considering both genetic and non-genetic risk factors. It is possible that the PLR may be impaired in association with some risk factors but not others, potentially indicating different pathways to neurodegeneration that could be distinguished using the PLR. If the PLR is impaired in individuals in specific risk groups for AD before symptoms of AD progress to a diagnosis, the PLR could then provide a quantitative measure to assist in predicting a person’s risk for developing AD in conjunction with a genetic, life event, and lifestyle analysis, and thus be used to identify potential patient-specific early interventions.

This review aims to bridge the gap in current literature that focuses on the impairment in AD patients, and to expand this to include the analysis of the PLR in groups displaying individual risk factors for the disease before a diagnosis is made. The aim is to investigate how these risk factors relate to one another, in addition to analyzing their relation to an altered PLR, if any, to determine whether the PLR is impaired in any at-risk groups for AD prior to diagnosing the disease. With the prevalence of pupillometry in current research, and the demand for a means of diagnosing preclinical AD with an inexpensive and accurate tool, analyzing the PLR of subjects who may be susceptible to developing the disease later in life could be a valuable area of research which, if successful, could lead to further advancements in the prevention of AD.

### 1.3. Goals

The specific objectives of this review are to provide an overview of the most prevalent AD risk factors (genetic and non-genetic), discuss pupillometry and the PLR, and investigate the relationship between AD risk factors and PLR.

In particular, this review focuses on research that has been done relating the PLR to specific lifestyle factors that have been linked to AD risk.

## 2. Alzheimer’s disease risk factors

There are many risk factors for AD and dementia, which can be split broadly into two categories: genetic risk factors, and non-genetic risk factors.

### 2.1. Genetic risk factors

Intrinsically, AD, specifically early onset AD, is often caused by mutations in one of three genes: amyloid precursor protein, presenilin 1, and presenilin 2 (van der Flier et al., 2011; Tzekov and Mullan, 2014). Late onset AD is not necessarily as predictable but can be indicated by inheritance of the ɛ4 allele of the APOE gene (Liu et al., 2013; Tzekov and Mullan, 2014). The inheritance of the APOE ɛ4 allele is the strongest genetic risk factor for AD – although only about 20–25% of the population carries one or more ɛ4 alleles, 50–65% of people diagnosed with AD carry the allele (van der Flier et al., 2011).

The inheritance of one or more ɛ4 alleles has implications on the age of onset of AD. Having at least one ɛ4 allele is associated with a reduced onset age for AD, and people with two ɛ4 alleles can develop AD up to 10 years earlier than those without the allele (van der Flier et al., 2011). Despite being associated with the age of onset of AD, it is not clear whether carrying the ɛ4 allele is also a risk factor for a faster progression of the disease once dementia has been reached. Contrary to the ɛ4 allele, the presence of the ɛ2 allele can help to reduce the risk of developing AD (van der Flier et al., 2011).

Using functional MRI, studies have shown that the ɛ4 allele moderates brain function (Suri et al., 2015). This moderation of brain function includes changes in white matter integrity and brain connectivity, and may make the brain more susceptible to age-associated pathological mechanisms such as amyloid beta accumulation (Reinvang et al., 2013). Further, this moderation is evident in young adults decades before any potential cognitive decline (Suri et al., 2015). However, other functional MRI studies using blood-oxygenation-level-dependent (BOLD) contrast have reported similar BOLD activity in both ɛ4 and ɛ2 carriers, despite the expectation that the high-risk ɛ4 carriers would have an opposite activation to low risk ɛ2 carriers; it is thus necessary to consider more than the functional MRI signal to determine the relationship between APOE, AD risk, and brain function (Suri et al., 2015).

### 2.2. Non-genetic risk factors

There are several extrinsic, life-event, or lifestyle-based, risk factors for AD that have been identified. Historically, dementia was not considered to be preventable or treatable; within recent years progress has been made to identify non-genetic risk factors for the disease and to collect information on preventing and managing the disease (Livingston et al., 2017). Although the underlying symptoms and illnesses with dementia may not be curable, the current understanding is that the progression and handling of the disease can be manageable when considering these non-genetic, lifestyle-based risk factors and factors that are considered to be protective against the disease, including aspects of diet, physical activity, and levels of cognitive reserve (Livingston et al., 2017; Silva et al., 2019).

Dementia is most common among adults aged 65 years or older, which is incidentally when age-related physical health problems and dementia co-occurring is common (Livingston et al., 2017). Additionally, these physical health problems often overlap with the lifestyle-based risk factors that increase the risk of dementia; an impaired mental and physical function may interfere with a person’s regular scheduling of things such as exercise and social interactions, all of which can further contribute to dementia risk (Livingston et al., 2017). Further, ethno-racial and socioeconomic factors can have an important impact on a person’s lifestyle, and so these factors must be considered as well and research conducted into dementia risk factors within individual populations cannot be considered adequate to apply to all people (Babulal et al., 2019; Livingston et al., 2020). As such, when considering some lifestyle-based risk factors and their specific contributions to dementia risk, it is necessary to consider the comorbidity of individual genetic, lifestyle, social, cultural, and economic risk factors, in dementia cases.

In 2017, The Lancet Commission published an in-depth analysis of the main, potentially modifiable, risk factors for AD (Livingston et al., 2017); this list was updated in 2020 with three additional factors identified (Livingston et al., 2020). This analysis sought to estimate the Population Attributable Factor (PAF), defined as the percentage reduction in new dementia cases over a given time if a specific risk factor were eliminated completely, for known modifiable risk factors for dementia (Livingston et al., 2017). The risk factors that were included in the PAF calculations were chosen by identifying risk factors listed in the UK National Institute of Health and Care Excellence (NICE) and US National Institute of Health (NIH) guidelines. Specifically, The Lancet Commission has identified 12 main categories for modifiable risk factors, through a systematic review and meta-analysis (Livingston et al., 2020). As will be shown throughout this review, these risk factors appear in a notable number of recent studies, and so these were taken to be the basis of this review’s focus. These risk factors, and their relative weightings in terms of the percentage of AD cases they cause, are categorized into early life (age < 45 years), midlife (age 45–65 years), and late life (age > 65 years; Livingston et al., 2020). These risk factors are shown in Table 1.

**Table 1:** Potentially modifiable risk factors for AD and their PAF, as calculated by Livingston et al. (2020).

Category	Risk factor	PAF
Early life, potentially modifiable	Less education	7%
Mid-life, potentially modifiable	Hearing loss	8%
	Traumatic brain injury	3%
	Hypertension	2%
	Alcohol consumption (greater than 21 units/week)	1%
	Obesity	1%
Later life, potentially modifiable	Smoking	5%
	Depression	4%
	Social isolation	4%
	Physical inactivity	2%
	Air pollution	2%
	Diabetes	1%
Risk unknown		60%

Although there are potentially other risk factors for dementia, these 12 main lifestyle/life event risk categories can be used to form a basis of factors to analyze the comorbidity of risk factors and to assess their relation to other biomarkers of AD. It is important to acknowledge however, that the evidence that has been collected by Livingston et al. about these AD risk factors is from high income countries, and thus these risks could differ in other countries and corresponding interventions may require modifications in specific environments (Livingston et al., 2020). In this review, we will be assessing these 12 lifestyle and life event categories with their relation to the parasympathetic/sympathetic pathways and to the pupillary light response, if any.

## 3. Pupillometry and parasympathetic/sympathetic pathways

### 3.1. Pupillary stimuli and measurements

**Figure 1:**
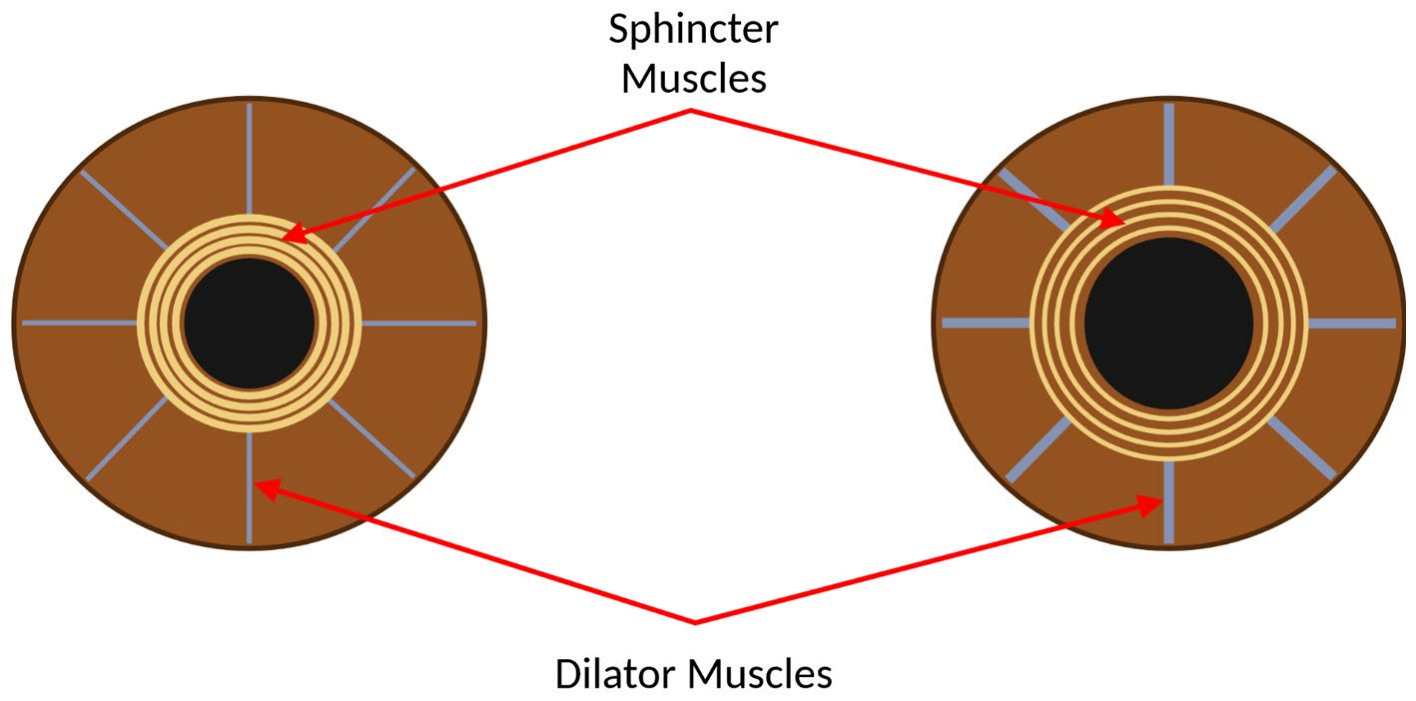
The eye, with the sphincter and dilator muscles labelled. Left: the eye with the pupil constricted. Right: the eye with the pupil dilated. This figure was created by SS using BioRender.com.

Pupillometry has been established as a promising means for assessing cognitive function, among other cerebral and bodily functions. Ultimately, the size and responsiveness of pupils in humans is controlled by the two main branches of the autonomic nervous system: the sympathetic and parasympathetic nervous systems, which control the dilator and sphincter muscles in the iris, respectively (Winn et al., 1994; Wang et al., 2016). Figure 1 shows the sphincter and dilator muscles on a constricted and dilated pupil. Further, pupillary constriction, accommodation, and vergence make up the near triad visual response, which enables a focused image, increased depth of focus, and binocular vision (Schor, 1992; Feil et al., 2017). To maximize the impact of using pupillometry to assess specific functions, namely parasympathetic and sympathetic functions, it is necessary to stimulate the pupil and measure concomitant pupil size changes with a measurement system that is best suited to measure these changes.

#### 3.1.1. Pupillary measurement systems

One concern when using pupillometry as a diagnostic tool is ensuring consistency of measurement across different individuals with different eye sizes, and these individual differences must be accounted for. Usually, these differences are accounted for by expressing values relative to a baseline. Many pupillary measurement systems assess relative changes in either the pupil size or the ratios between the pupil and iris. One measurement method to correct for differences in eye sizes between subjects, as described by Fotiou et al., is to use the ratios between the current pupil radius (P), the baseline pupil radius (B), and the iris radius (I) at each measurement point in time (Fotiou et al., 2000). Fotiou et al. tested the use of the pupil-to-baseline pupil ratio (P/B) to determine which would be most suitable to produce the most reliable results, and the result of their study was that the pupil-to-iris method was preferred as the baseline pupil size is difficult to keep consistent even within the same individual, whereas the iris size is a stable anatomical marker (Fotiou et al., 2000). The pupil-to-iris (P/I) ratio is a popular method that has been used by multiple researchers (Sacks and Smith, 1989; Lanting et al., 1990; Fotiou et al., 2000).

When making pupillary measurements, it is also important to identify an appropriate time and sampling frame for measurements. The pupil is constantly changing size due to small oscillations of the pupil, known as hippus (Winn et al., 1994). Due to these continuous changes, it is not reliable to take a single measurement of the pupil size. For the most accurate measurements, the pupil size should be sampled frequently over a suitable time period to obtain reliable measurements that can account for these minor oscillations (Winn et al., 1994).

One way in which continuous measurements can be made is by using automated measurements through deep learning techniques. One web app that uses deep learning for translational and real-time pupillometry is MEYE, developed by Mazziotti et al. (2021). To make the pupillometry measurements, they applied random rotation, cropping, horizontal and vertical flipping of images, in addition to random brightness, contrast, and sharpness changes to train the model (Mazziotti et al., 2021).

#### 3.1.2. Pupillary stimuli

There are many ways to stimulate pupillary changes – each of which has different diagnostic purposes. Pupillometry studies vary widely in their stimuli, and without a standard methodology used across all pupillometry-related studies, there is a challenge presented in comparing the results of these studies (Kelbsch et al., 2019). Despite this, studies using similar methods can still be compared.

Many studies have shown how cognitive processes can cause pupillary changes – specifically, emotional arousal, interest, and task difficulty (Hess and Polt, 1960; Hess, 1965; de Winter et al., 2021). When the cognitive task demand is increased over time, the pupil dilates following this stimulus, and then constricts when the subject has less difficulty with the cognitive task at hand (Siegle et al., 2003; Steinhauer et al., 2004). Further, when performance is sustained during a difficult task, this is modulated by the cortical inhibition of the parasympathetic pathway located at the oculomotor nucleus (Steinhauer et al., 2004).

Another stimulant that has been used in pupillometry studies, specifically when assessing potential neurodegeneration, is a dilute solution of tropicamide. Administering tropicamide can block the parasympathetic sphincter muscle, which impacts the pupillary reaction (Steinhauer et al., 2004). Scinto et al. show that AD patients, or probable AD patients who have not yet been diagnosed with AD, have a more pronounced pupillary reaction and hypersensitivity to a dilute solution of tropicamide when compared to normal controls, suggesting that their parasympathetic sphincter muscle works abnormally when compared to normal controls (Scinto et al., 1994). Further, Higuchi et al. show that subjects with the APOE ɛ4 allele have a more hypersensitive response to tropicamide (Higuchi et al., 1997). However, not all studies agree on the effects of tropicamide on pupillary reactions in AD patients – Granholm et al. found that AD patients did not differ significantly in pupillary responses to tropicamide when compared with cognitively normal controls (Granholm et al., 2003). Although Granholm et al. attempted to use similar methods to Scinto et al., including a 0.01% dilute solution of tropicamide, there may have been variability between the subject groups of the studies that could account for different results. Granholm et al. note that ethnicity, eye color, age, and background luminance may be important factors that could impact the pupillary response to tropicamide, and so their study tested both light and dark conditions and had subjects similar in age, gender, eye color, and ethnicity (Granholm et al., 2003); in contrast, Scinto et al. did not report ethnicity, eye color, or background luminance in their methods, which could explain their conflicting results with Granholm et al. (Scinto et al., 1994). Additionally, neither Scinto et al. nor Granholm et al. reported on genetic features of their subjects, and since those with the APOE ɛ4 allele have a hypersensitive response to tropicamide, not accounting for this could also explain differences in results. The pupillary response to different light conditions is also an important area of study. The pupillary darkness reflex, and the recovery time for the pupillary light response, are controlled primarily by sympathetic activation, whereas the amplitude and latency of the pupillary light response is controlled by parasympathetic activity (Prettyman et al., 1997). As such, assessing the pupillary light and darkness responses, which can be done by changing light conditions, is a useful method for stimulating the pupillary changes (Ellis, 1981; Prettyman et al., 1997; Steinhauer et al., 2004).

Different light sources can be used to measure and assess the PLR, and the response can be influenced by the duration, spectral composition, and intensity of the light used as a stimulus (Hall and Chilcott, 2018). The differences in stimuli determine which photoreceptor classes are activated - the rod responses, cone responses, or melanopsin-driven ipRGCs (Kelbsch et al., 2019). Infrared pupillometry, for example, is a method that is particularly useful when assessing the PLR, which involves stimulating the pupil with an infrared light source and then observing the response on an infrared sensor (Ellis, 1981). Automated infrared pupillometry can also provide a measurement of the PLR that is more reliable than using a manual flashlight to examine the PLR (Romagnosi et al., 2020). Additionally, chromatic pupillometry, which involves protocols using light stimuli at different wavelengths to isolate the contributions of single photoreceptors, is a method used to characterize melanopsin retinal ganglion cells which are photoreceptive and are most sensitive to blue light at 480 nm (Romagnoli et al., 2020).

## 4. Comorbidity of factors affecting pupillary response and risk to Alzheimer’s disease

The following subsection will review the prevalent risk factors for AD and their relation, if any, to an impaired pupillary response. The comorbidity of these risk factors will also be assessed to evaluate the individual impact on the pupillary response from the individual and combined risk factors, and confounding factors will be identified and reviewed.

### 4.1. Overview of impaired pupillary responses in Alzheimer’s disease

Before assessing the pupillary responses in cases involving specific risk factors to AD, it is important to assess the pupillary responses in AD patients. Much of the research into pupillary responses in AD does not assess specific risk factors and considers the impact of dementia on the PLR in general, and so this section aims to review some of this broader research before assessing individual risk factors.

There is precedence for the study of impaired pupillary responses in various physical and mental conditions including AD, to study disturbances in the parasympathetic responses relating to the pupil (Fotiou et al., 2008). In patients with AD, changes relating to vision are some of the first symptoms that impact patients (Chang et al., 2014). Potential ocular biomarkers for AD include visual acuity, contrast sensitivity, pupil reaction, color vision, visual field, motion perception, ocular motor function, and stereopsis (Chang et al., 2014; Beltrán et al., 2018). When assessing the pupil reaction in AD, Chang et al. suggest that changes are expected in the pupillary light and dark reflexes (Chang et al., 2014), the former of which will be reviewed in the following section.

A summary of AD risk factors and their impact on the PLR is shown in Table 2, which indicates whether an impact was identified or not, and which section each risk factor is discussed in.

**Table 2:** Summary of AD risk factors and their impact on the PLR.

Risk factor	Impact on the PLR (note: + indicates authors identified an impact on the PLR; − indicates authors did not identify an impact on the PLR; ? indicates authors identified a potential, but unconfirmed, impact on the PLR.)	Section discussed in
Genetics	?	4.2.1
Level of education	−	4.2.2
Hearing loss	?	4.2.3
Traumatic brain injury	+	4.2.4
Hypertension	+ (intracranial and ocular hypertension) ? (general hypertension)	4.2.5
Alcohol consumption	+	4.2.6
Obesity	−	4.2.7
Smoking	?	4.2.8
Depression	+	4.2.9
Social isolation	−	4.2.9
Diabetes	+	4.2.10
Physical inactivity	−	4.2.11

#### 4.1.1. Pupillary light response in Alzheimer’s disease

**Figure 2:**
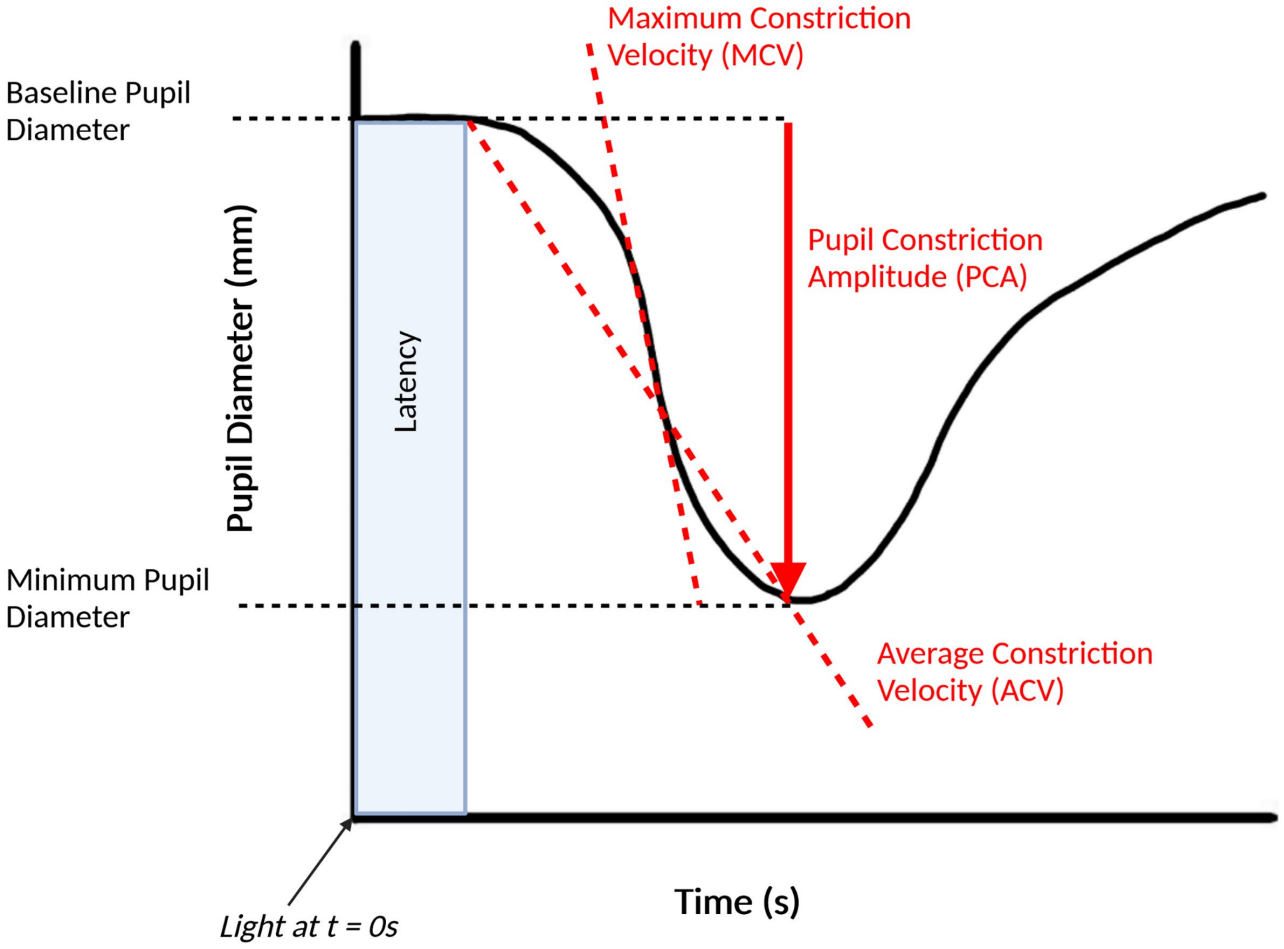
Pupillary light response plot, with commonly extracted features labelled. This figure was created by SS using BioRender.com.

There are a multitude of studies that investigate the pupillary light response in AD, each of which use specific combinations of stimuli and measurement systems. Figure 2 shows some of the commonly used features of the PLR that are assessed in AD studies. One area of study involves assessing the resting pupil size of AD patients and comparing this to cognitively normal controls. When comparing the two groups, Kawasaki et al. found that the baseline pupil size in room light was significantly smaller in the AD group (Kawasaki et al., 2020). Frost et al. found similar results, finding a smaller resting (after a 2-min dark adaptation) and minimum pupil size after stimulus in the AD group (Frost S. et al., 2013). Finally, Prettyman et al. found a smaller resting pupil size in the AD group compared to the control group, which they hypothesize could be caused by a sympathetic deficit caused by the loss of neurons in the locus coeruleus in AD (Prettyman et al., 1997).

However, not all studies have replicated the studies led by Kawasaki and Prettyman. Fotiou et al. found no significant difference in baseline pupil size between the AD group and cognitively normal controls (Fotiou et al., 2007b). Additionally, Ferrario et al. found that the baseline pupil size was notably higher in the AD group than in the control group (Ferrario et al., 1998). These discrepancies could potentially be due to different root causes of AD, which may determine whether the PLR parameters such as baseline pupil size are impacted. As such, more research is required into an analysis of baseline pupil size in AD to obtain an accurate and reliable conclusion.

Another area of research is an analysis of the velocity and acceleration of pupil constriction or dilation in AD patients compared against cognitively normal groups. Generally, most studies of pupillometry have found a reduced maximum constriction velocity (MCV) and maximum constriction acceleration (MCA) in AD cohort when compared to healthy controls, consistent with a hypothesized parasympathetic deficiency, and have been proposed as the most accurate pupillometric parameter for differentiating AD groups from healthy controls (Chougule et al., 2019). Fotiou et al. observed significantly lower values for both MCV and MCA in the AD group when compared to the controls and noted that MCA was the best parameter to separate AD patients from healthy controls, with MCV in a close second (Fotiou et al., 2007b). Frost et al. similarly found reduced values for MCV and MCA in AD groups but found that MCV was the best method for classifying AD groups from controls (Frost et al., 2017). Other studies by Fotiou et al. and Frost et al. have reported reduced MCV and MCA in AD groups (Frost S. et al., 2013; Fotiou et al., 2015).

Prettyman et al. found that patients in the AD group had a reduced recovery time when compared to the control group, however they caution that this response, in conjunction with a reduced amplitude of the response, could be due to a saturation effect at the response floor, which could make it difficult to draw conclusions about how much the parasympathetic innervation of the iris plays a role in AD (Prettyman et al., 1997).

In contrast to most studies, Ferrario et al. found that the average values for MCA were higher in the AD group than in the control group and did not find a statistical difference between the MCV of the AD and control groups (Ferrario et al., 1998). Although most studies agree that MCV and MCA are reliable pupillometric markers to differentiate AD patients from healthy controls, this is not always the case – this could be due to different risk factors for AD resulting in different disease endotypes.

Other pupillometric parameters that are used in pupillometry studies involving AD are the pupil constriction and pupil dilation amplitudes. Most studies have found that AD groups have a decreased pupil constriction amplitude (PCA) when compared to healthy controls, including those led by Fotiou et al. (2007b), Frost S. et al. (2013), Frost et al. (2017), and Chougule et al. (2019). Prettyman et al. found a reduced PCA in the AD group, but caution that just as with the reduced recovery time, that this could be due to a “floor effect” which makes it difficult to conclude that a parasympathetic innervation of the iris in AD causes these effects (Prettyman et al., 1997). Granholm et al. found that the peak PCA was significantly reduced in AD groups when compared to healthy controls but was also reduced in Parkinson’s disease patients and that there was not a significant difference between the AD and Parkinson’s disease groups, which they suggest means that this test is sensitive to AD but does not have adequate specificity (Granholm et al., 2003).

As with the other pupillometry parameters, not all studies have found a decreased PCA in AD patients. Ferrario et al. did not find a significant difference between the PCA of AD and control groups (Ferrario et al., 1998). Similarly, Kawasaki et al. did not find any significant difference in PCA when subject to various intensities of colored light in the AD group (Kawasaki et al., 2020). Van Stavern et al. did not find any significant difference between a preclinical AD group and the PCA (Van Stavern et al., 2019). As such, more research should be done to assess the validity of the PCA as a pupillometric indicator for AD.

There have been some significant relationships found between AD patients and their pupillary light responses, including their resting pupil diameters, their MCV and MCA, and their PCA. However, not all these relationships show the same reproducibility as shown by some contradictory studies. Additionally, some of these relationships are also seen in other neurodegenerative diseases, which reduces the specificity of only using these PLR metrics to separate AD patients from others. Many of these differences may be due to notable differences in the inclusion and exclusion criteria of subjects across studies, including ethnicity, sex, genetics, comorbidities and other demographics and disease endotypes. More information, including any underlying health conditions or other risk factors, should be collected from patients to be able to separate the pupillary response impacts of AD alone from the impacts from other factors, and to account for any comorbidities that may impact the response.

### 4.2. Risk factors and pupillary light response impairments

The pupillary light response appears to be impacted in AD patients; however, this measure alone lacks the required specificity to separate AD patients from healthy controls or those with other diseases. This section will review the prominent risk factors for AD and their impacts, if any, on the PLR. The aim will be to separate the individual impacts on the PLR in AD patients who may have these underlying conditions or risk factors, and to assess the comorbidity of these conditions in relation to the PLR.

#### 4.2.1. Genetics

Before investigating the impact of various lifestyle and preventable risk factors for AD on the PLR, genetic risk factors will be explored – specifically, the APOE ɛ4 allele, and genetic factors involved in autosomal dominant AD (ADAD). There have been several studies investigating changes in the pupillary responses in subjects with genetic susceptibilities to AD – specifically when assessing the pupillary responses to light and to tropicamide.

ADAD is a rare form of AD that affects carriers with specific gene mutations, which can occur in people as young as 30 years old. The gene mutations involved in this genetic disorder primarily involve amyloid precursor protein (AβPP), presenilin 1 and presenilin 2, and mutation carriers progress to AD with 100% certainty (Frost S. M. et al., 2013). Frost et al. investigated the pupil flash responses in mutation carriers (specifically in the APP 693 mutation at position 22 of the amyloid-beta fragment, APPGlu693Gln) and compared them to non-carriers, all within a single family. They found that the 75% recovery time was larger in the mutation carrier group and the percentage recovery 3.5 s post-stimulus was smaller in the mutation carrier group – both parameters were found to provide perfect classification of mutation carriers against non-carriers in the cohort, showing that the pupil flash response can be used in AD cases outside of the sporadic AD classification (Frost S. M. et al., 2013).

The pupillary response to tropicamide has been investigated in several studies relating to AD. A study by Higuchi et al. found that cognitively normal subjects with the APOE ɛ4 allele had a greater increase in pupil size after tropicamide-induced changes, which they suggest shows that this hypersensitivity can be seen in APOE ɛ4 carriers before the onset of AD (Higuchi et al., 1997). These findings agree with a study by Turana et al., which found that subjects with the APOE ɛ4 allele had the highest pupillary hypersensitivity response when a drop of 0.01% tropicamide was put on their eye, when compared to the other subjects, and suggested that a combination of biological and clinical markers is required to increase the positive predictive value towards amnestic mild cognitive impairment cases (Turana et al., 2014).

It is evident that there are some genetic factors that are associated with different pupillary responses. However, more research should be done on the PLR in APOE ɛ4 carriers and carriers of specific genetic mutations to make any conclusions about how these factors influence the PLR.

#### 4.2.2. Level of education

A lower cognitive reserve leads to vulnerability to cognitive decline (Valenzuela and Sachdev, 2006), as cognitive reserve assists in maintaining brain function (Valenzuela, 2008). Individuals with a higher cognitive reserve have a later onset of cognitive functions being impacted by AD or age-related pathology, as their higher cognitive reserve can tolerate more pathology (Stern, 2012). Thus, increasing a person’s cognitive reserve can assist in preventing dementia. The level of education that a person receives, and their occupational status, are both factors that contribute to increasing the brain’s cognitive reserve (Valenzuela and Sachdev, 2006). Cognitive ability increases with education before plateauing in late adolescence with few further improvements with education after an age of 20 years (Kremen et al., 2019) – thus, cognitive stimulation is especially important in early life to assist in building cognitive reserve. Stern et al. also found that participants in low occupational levels throughout their lifetimes, based on the United States census categories, have a greater risk of developing dementia (Stern, 2012). Other cognitive activities in adulthood, including reading, playing games, playing music and creating art, speaking multiple languages, and participating in leisure activities also assist in maintaining cognition (Valenzuela and Sachdev, 2006; Stern, 2012).

To the authors’ knowledge, no study has published any results that directly link the subject’s level of education or cognitive reserve with their pupillary responses, either to light or another stimulus. Research should be done to consider the direct impacts of education and cognitive reserve on the PLR both dependent and independent of AD to potentially aid in diagnosis, given that an increase in cognitive reserve can delay the onset of symptoms of AD and make a diagnosis more challenging (Stern, 2012).

#### 4.2.3. Hearing loss

Hearing loss at any scale, including mild hearing loss, increases the long-term risk of cognitive decline and dementia (Lin et al., 2011a,b; Gallacher et al., 2012; Kiely et al., 2012; Gurgel et al., 2014; Amieva et al., 2015; Deal et al., 2015, 2017; Fritze et al., 2016). In many cases, hearing loss can predate and predict a clinical diagnosis of dementia (Bowl and Dawson, 2019), and auditory scene processing deficits could be considered a functional marker for AD pathology (Johnson et al., 2021). According to Livingston et al., hearing loss had the highest population attributable fraction of potentially modifiable risk factors for dementia, and with every 10 dB of reduction in hearing a decrease in cognition is found, potentially due to reduced cognitive stimulation (Livingston et al., 2020). However, these findings are only consistent with people who do not use hearing aids – hearing aid use is one of the largest factors that can protect against the onset of dementia (Livingston et al., 2020).

Previous functional MRI (fMRI) studies conducted at resting state have shown a reduction in spontaneous neural activity in hearing loss patients which correlated with a reduction in cognitive performance (Ponticorvo et al., 2019). There are several possible mechanisms to explain the relationship between hearing loss and dementia, which are defined by Griffiths et al. as: common pathology affecting the cochlea and ascending pathway (causing hearing loss) and the cortex (causing dementia); impoverished environment causing decreased cognitive reserve; a requirement for increased cognitive resources for listening; the interaction between brain activity related to auditory cognition and dementia pathology (Griffiths et al., 2020).

General pupillary responses arising from an increase in mental or cognitive effort have been investigated in hearing loss subjects (Kramer et al., 1997; Zekveld et al., 2011, 2020). However, in their 2016 systematic review, Wang et al. did not identify any results for studies directly linking the pupil light reflex and hearing impairment (Wang et al., 2016). However, they did investigate hearing impairment and its associations with the parasympathetic response, which has been shown to be linked to the PLR (Wang et al., 2016). Hasson et al. found a negative correlation between hearing problems and parasympathetic activity and associated an increase in hearing problems with a decreased ability to “unwind” or recover from the stress due to diminished parasympathetic activity (Hasson et al., 2009). Mackersie et al. found that subjects with hearing loss had greater stress-related autonomic nervous system activation and noted that an important aspect of a stress response could include activation of the sympathetic branch and suppression of the parasympathetic branch (Mackersie et al., 2015).

No direct relation between the PLR and hearing ability or loss was found in the search conducted, and minimal evidence was shown to link a decreased parasympathetic response to hearing loss. Despite this, as the PLR is governed by sympathetic and parasympathetic activity, this potential link between hearing loss and a decrease in parasympathetic activity should be explored in further research, using the PLR as a metric.

#### 4.2.4. Traumatic brain injury

Traumatic brain injury (TBI), including mild and severe injuries, is a known risk factor for AD (Perry et al., 2016; Fann et al., 2018). In particular, a single, severe, TBI is associated with widespread hyperphosphorylated tau pathology in both humans and mouse models (Zanier et al., 2018). The risk of developing AD due to TBI increases with both the severity of the injury and the number of injuries sustained, and the risk of dementia is stronger closer to the time that the injury occurs which can lead to early-onset AD in some people (Fann et al., 2018). Notably, those with a higher occupational risk for head and brain injuries are more likely to develop AD as a result of their increased likelihood of these injuries (Barnes et al., 2018; Mackay et al., 2019; Yaffe et al., 2019). Many athletes including boxers, American football players, ice hockey players, soccer players, rugby players, and wrestlers, in addition to military veterans, have had associations with chronic traumatic encephalopathy (CTE), a progressive neurodegenerative disease which is associated with repetitive TBI experienced in sports and military activity and is a risk factor for dementia (Stein et al., 2014).

There have been several studies investigating the PLR in TBI subjects, through several different methods of classifying TBI. A common consequence of TBI is an increase in intracranial pressure (ICP), which is one such parameter used to characterize TBI (Noble, 2010). Chen et al. used an algorithm to characterize the pupillary response relating to ICP, called the Neurological Pupil index (NPi), which takes in common parameters of the PLR including the pupil’s minimum and maximum sizes, constriction percentage and velocity, and dilation velocity (Chen et al., 2011). They found that subjects who had decreased PLRs had higher peaks of intracranial pressure, using the NPi to characterize the pupillary responses (Chen et al., 2011). Another means of assessing TBI is through the Glasgow Coma Scale (GCS), which can be used to measure the neurologic status of patients, with more severe brain injuries being classified as 8 or less on this scale, and mild to moderate brain injuries being classified as 9 or more (Park et al., 2015). Park et al. found that diminished PLRs were associated with a lower GCS score and found that the initial NPi value of the group of subjects receiving a “poor” prognosis was lower than the group with a “favorable” prognosis, demonstrating the potential for the PLR to be used in diagnosing and classifying TBI severity (Park et al., 2015).

Several other studies have shown direct links between TBI and the PLR, showing that multiple parameters of the PLR are reduced in magnitude after TBI, particularly when using monocular test measurements (Truong and Ciuffreda, 2016; Oshorov et al., 2021). Most studies assess the PLR in the short term after TBI occurrence and do not follow-up on long term PLR changes, although Truong et al. found PLR impairments in mild TBI patients in the chronic recovery phase (greater than 45 days post-injury) when compared to normal controls (Truong and Ciuffreda, 2016). As such, there is sufficient evidence to show that TBI is associated with noticeable changes in the PLR, at least in the short term. More research should be done to investigate how these changes are affected in the long term.

#### 4.2.5. Hypertension

Hypertension, specifically persistent hypertension in midlife, is associated with an increased risk for dementia in later life. An elevated systolic blood pressure in midlife has been shown to increase dementia risk, with the risk increasing if this hypertension continues later in life (McGrath et al., 2017). A potential mechanism for how this contributes to dementia risk is through alterations of regulatory mechanisms of the cerebral circulation, which compromise the blood supply to the brain (Faraco and Iadecola, 2013). Additionally, midlife hypertension is associated with reduced brain volumes and an increased white matter hyperintensity volume (Lane et al., 2019). However, this risk can be reduced when anti-hypertensive medications are taken (Ding et al., 2020).

Hypertension has been linked to an altered PLR in several studies, however most studies focus on either intracranial hypertension or ocular hypertension. Grozdanic et al. have found, in separate studies, that the PLR is reduced in rats after acute elevation of intraocular pressure (Grozdanic et al., 2003, 2004). In human subjects, reduced amplitudes of the PLR have been associated with increased intracranial pressure and in intracranial hypertension including idiopathic intracranial hypertension and is often used to monitor neurocritical care patients (Taylor et al., 2003; Chen et al., 2011; Park et al., 2016; Jahns et al., 2019; Romagnosi et al., 2020).

Importantly, hypertension can affect the autonomic nervous system, which is characterized by sympathetic and parasympathetic activity. Multiple studies have shown that patients with borderline hypertension display an increase of sympathetic activity (Julius et al., 1971, 1991; Anderson et al., 1989; Mancia and Grassi, 2014), and a decrease of parasympathetic activity (Julius et al., 1971; Mancia and Grassi, 2014). Although not many studies have directly evaluated the effects of general hypertension on the PLR, its link to the sympathetic and parasympathetic activity shows that further research should be conducted in this area to investigate a link between the PLR and general hypertension.

#### 4.2.6. Alcohol consumption

Heavy drinking has been associated with cognitive impairment and dementia (Rehm et al., 2019), however due to its complex entanglement with sociocultural and health-related factors, it is challenging to fully understand how alcohol alone contributes to dementia risk (Livingston et al., 2020). Venkataraman et al. suggest that alcohol misuse, such as binge drinking or chronic alcohol use, could lead to neuroinflammation and neuronal cell death, which could be a mechanism for how alcohol consumption increases AD risk (Venkataraman et al., 2017). Additionally, higher alcohol consumption has been associated with an increased risk of hippocampal atrophy, which is considered a specific marker of AD (Topiwala et al., 2017). Regardless of the mechanisms involved, moderating or reducing alcohol intake can reduce the risk of AD (Rehm et al., 2019).

The parasympathetic response may be impaired in alcoholics, due to lesions in the parasympathetic supply (Tan et al., 1984). This has been shown to manifest as an impaired PLR when comparing alcoholics to non-alcoholics (Rubin, 1980; Chida et al., 1998). Rubin also compared alcoholics who abstained from drinking 1 month prior to the study to alcoholics who did not abstain and found that both groups had an equally defective rate of pupillary contraction, but the alcoholic drinkers had a slower rate and amplitude of dilation, showing that alcoholics demonstrate an impaired parasympathetic outflow regardless of their drinking activity, but that the sympathetic deficiency is dependent on whether the alcoholic abstains from drinking for an extended period of time (Rubin, 1980).

Changes in the PLR are not only observed due to consistent alcohol consumption over an extended period but are observed during the act of consuming alcohol or while a person is actively drunk. Short term alcohol consumption leads to dilated pupils and slower pupillary reactions (Dhingra et al., 2019). Studies have shown that the PLR may be a good measure to classify a person’s current inability to work or drive due to alcohol consumption or sleep deprivation (Jindou et al., 2010; Kaifie et al., 2021). As such, it is important to separate the instantaneous impacts of alcohol consumption from the more prolonged impacts from alcoholism or heavy drinking on the PLR, and more research should be done to separate the two.

#### 4.2.7. Obesity

An increased body-mass index (BMI), specifically in the obesity-defined range, is associated with an increased risk of dementia (Albanese et al., 2017). Additionally, increased adiposity is related to AD, potentially due to increased vascular stress, however the exact mechanisms for this are still unknown (Luchsinger and Gustafson, 2009). Further, the risk of dementia has been shown to vary with the age of onset of obesity, with a higher risk associated with younger adults with obesity when compared to adults who only develop the condition later in life (Wotton and Goldacre, 2014). While there is data that supports the claim that weight loss in obese and overweight adults is associated with improvements in performance across multiple cognitive domains (Veronese et al., 2017), according to Livingston et al., there is no data specific to the long-term effects of weight loss in overweight and obese adults in lowering dementia risk (Livingston et al., 2020).

There is limited research that assesses the links between obesity and the PLR, and the limited research presents conflicting results. Baum et al. found a decreased PLR with an increased BMI in children and adolescents (Baum et al., 2013). Blüher et al. assessed changes in the PLR of obese children, after exercise and lifestyle interventions were made to decrease BMI and found that reductions in BMI were associated with a higher dilation velocity, higher relative light reflex amplitude, and higher constriction velocity (Blüher et al., 2015). Within a group of healthy adults with a range of BMIs ranging from normal to obese, Segal et al. found that those with a higher BMI also had a higher average dilation velocity post-stimulus and concluded that BMI levels positively correlate with sympathetic activity (Segal et al., 2022). When investigating sets of identical twins who had different BMIs (obese and non-obese classifications), Piha et al. did not find significant differences in heart rate, blood pressure, or pupillary responses between the obese and non-obese twins and concluded that neither sympathetic nor parasympathetic responsiveness is significantly affected by obesity and instead is affected significantly by genetic factors (Piha et al., 1994). These differences may be due to the different subject selection for these studies, including variability in age and genetics. Additionally, these changes could be due to other risk factors or comorbidities that may accompany obesity, such as physical activity levels and other lifestyle aspects.

No other significant study comparing obesity, BMI, or other weight-related factors to the PLR was found in the search. The studies that have been published to date used different age and genetic groups and have all presented different results, and so no conclusion can be made. Further research should be done with broad ranges of age and genetic groups to investigate any potential relationship between obesity and the PLR.

#### 4.2.8. Smoking

Smokers are at a higher risk of developing dementia when compared to non-smokers (Ott et al., 1998; Anstey et al., 2007; Cataldo et al., 2010; Rusanen et al., 2011). However, smokers have a higher risk of premature death which could occur before their age of dementia onset, so these competing risks may introduce biases and discrepancies in the association between smoking and dementia risk (Debanne et al., 2007; Chang et al., 2012). Regardless, not smoking can increase life expectancy and health, and stopping smoking can reduce the dementia risk (Choi et al., 2018). Exposure to smoke through second-hand smoke is also associated with more memory deterioration (Pan et al., 2018), although limited literature exists in this specific area (Livingston et al., 2020).

To the authors’ knowledge, no study has published any results that directly link long-term nicotine smoking to an impaired PLR. However, there have been studies that have investigated the relationship between nicotine and smooth muscle function. Studies have shown that nicotine may act on vascular smooth muscle and induce vascular relaxation in rats (Xu et al., 2015) or vascular contraction or relaxation in humans (Hanna, 2006). Further, nicotine may alter vascular smooth muscle cell phenotypes (Yoshiyama et al., 2014; Wang et al., 2019). Because the function of vascular smooth muscle cells may be a biomarker for AD, and these vascular smooth muscle cells may undergo phenotypic transitions in AD (Hayes et al., 2022), this supports the need for further research in this area.

Additionally, there have been studies linking smoking and intraocular pressure. Mansouri et al. found that chronic long-term smokers had a higher mean intraocular pressure than non-smokers (Mansouri et al., 2015). Similarly, Lee et al. found that current smokers had a slightly higher mean intraocular pressure than the non-smokers in their study (Lee et al., 2003). Although not directly linked to the PLR, this change in intraocular pressure should be investigated further to determine whether it, in turn, causes a change in the PLR in smokers.

#### 4.2.9. Depression

Having depression is associated with an increased risk of dementia and AD (Caraci et al., 2010; Dotson et al., 2010). Depression is also part of the dementia prodrome and can be seen in the early stages of the disease, and thus there has been debate as to whether depression is only a symptom of dementia, or if it is an independent risk factor for dementia (Dotson et al., 2010). The mechanisms involving depression and AD are likely to be multifactorial and may include vascular and neuropathological mechanisms (Chi et al., 2014). Some molecular mechanisms, such as chronic inflammation, are common in the pathogenesis of both major depression and AD (Caraci et al., 2010). Livingston et al. have not found conclusive evidence for the difference between treated and untreated depression regarding the risk of dementia (Livingston et al., 2020); however, the use of antidepressants has been shown to improve amyloid beta clearance (Sheline et al., 2014).

Some studies have investigated the PLR in subjects with depression, in various capacities. The PLR has been shown to be altered in patients with major depressive disorder (MDD) when compared to controls (Miller et al., 2021). Mestanikova et al. found that the PLR was diminished in the left eye of adolescent girls with depression, but not in the right eye (Mestanikova et al., 2017). Berman et al. found that the PLRs were diminished in depressed patients both with and without a seasonal pattern, when compared to healthy controls (Berman et al., 2018). Further, Fountoulakis et al. found that subjects with depression had a shorter latency for pupil constriction post-illumination, when compared to healthy controls, which suggests a norepinephrine hypoactivity in melancholic depression (Fountoulakis et al., 1999).

Some researchers have assessed how the PLR can be impacted by the conditions that the depressed subjects are under. Bar et al. found that the PLR was impacted by antidepressant use – they found that acutely depressed patients who had not taken antidepressants did not differ significantly in PLR parameters, other than relative amplitude, compared to healthy controls, although those taking antidepressants had significant changes in their parasympathetic function (Bär et al., 2004). Feigl et al. investigated how the mean daylight exposure could impact the PLR in subjects with mild and moderate non-seasonal MDD but found no significant differences between the MDD subjects and healthy controls regardless of the daily and hourly light exposure including recommended light therapy that is recommended for MDD patients (Feigl et al., 2018). However, Laurenzo et al. found that in addition to the PLR being impacted in MDD subjects when compared to healthy controls, MDD subjects displayed reductions in the post-illumination pupil response to high-intensity blue light, which was less pronounced in months with fewer daylight hours (Laurenzo et al., 2016). This may be due to the difference in methods and stimuli used or due to other differences in subject inclusion criteria, More research should be done to investigate impaired PLRs in depressed subjects of various ages and under various conditions.

Although considered to be a separate risk factor from depression, aspects of social isolation have several similarities with depression. However, to the authors’ knowledge, no study has published any results that link the subject’s level of social engagement with their pupillary responses, either to light or another stimulus, and thus the social isolation risk factor has not been further explored in this review.

#### 4.2.10. Diabetes

Having diabetes is a significant risk factor for dementia and AD (Baglietto-Vargas et al., 2016). It is thought that diabetes, specifically type 2, could increase the risk through insulin resistance, impairing glucose metabolism in the brain (Liu et al., 2009). Dementia risk is higher with increased duration and severity of diabetes, but the effects of diabetic medications on dementia outcomes or cognition are unclear (Livingston et al., 2020). It is generally agreed that type 2 diabetes is a risk factor for the future development of dementia, however specific treatment for diabetic control has not been shown to decrease dementia risk (Areosa Sastre et al., 2017).

There are extensive studies that have assessed the relation between diabetes and the eye, including several aspects of the PLR. Lanting et al. found that overall, the diabetic patient groups studied had a higher PLR latency when compared to healthy controls and normal values and claims that this represents parasympathetic dysfunction (Lanting et al., 1990, 1991). A study by Bista Karki et al. also supported the concept of parasympathetic dysfunction in diabetic patients, with results that showed that the diabetic subjects had a lower maximum and mean constriction velocity, lower constriction amplitude, and a lower relative reflex amplitude when compared to the healthy controls (Bista Karki et al., 2020). Ishibashi et al. subjected diabetics and healthy controls to both red and blue light, and found that with both light colors, the pupil constriction was slower and less pronounced in the diabetic group when compared to the healthy control group (Ishibashi et al., 2017). Karavanaki et al. compared diabetic children with healthy children and found that the diabetic group had impaired pupillary adaptation in the darkness (Karavanaki et al., 1994). Several studies that found a reduced PLR in diabetic patients when compared to healthy subjects, with further reductions when comparing diabetics with autonomic neuropathy and without, suggested that pupillometry could help to identify diabetic autonomic neuropathy (Yang et al., 2006; Ferrari et al., 2007; Feigl et al., 2012).

Not all studies support the claim that the PLR is significantly different between diabetic and non-diabetic subjects. Lerner et al. noted that although there were some differences in pupillometry values, that most had poor accuracy as a screening tool due to inadequate specificity and sensitivity (Lerner et al., 2015). Hreidarsson and Gundersen found that in type 1 diabetics who had a normal or near-normal sensory pathway, there was no significant difference in latency or other PLR parameters when compared to healthy controls with the same pupil size, and only a minor reduction in response amplitude (Hreidarsson and Gundersen, 1985).

Overall, there are multiple studies that show a reduction in the PLR among diabetic subjects when compared to healthy controls. Pupillometry has been suggested as a diagnostic tool for the monitoring of diabetes progression, specifically when assessing the development of certain side effects including autonomic neuropathy. However, there is a potential confound with diabetic retinopathy, where pupillary abnormalities may precede a diabetic retinopathy diagnosis (Bista Karki et al., 2020), and thus it is difficult to know whether any impairments to the PLR are due to the retina or due to nervous system defects. Additionally, there could be confounding effects with comorbidities that may exist with diabetes, which could also be different in type 1 and type 2 diabetes cases.

#### 4.2.11. Physical inactivity

Physical inactivity is a risk factor for dementia, and older adults who exercise regularly have a better chance of maintaining cognition (De la Rosa et al., 2020). Being physically active is considered a protective factor against cognitive decline (Sofi et al., 2011; Hersi et al., 2017). Livingston et al. highlight that although physical inactivity is considered a separate risk factor for dementia, there are several overlaps between physical activity and other risk factors such as obesity and diabetes, and confounding factors exist with age, sex, social class, and cultures (Livingston et al., 2020). To the authors’ knowledge, no study has published any results that link the subject’s level of physical activity with their pupillary responses, either to light or another stimulus, and thus this risk factor will not be explored further in this review.

### 4.3. Confounding factors

As was highlighted earlier, pupillary responses can be evoked due to a variety of factors and stimuli. Outside of the non-genetic and lifestyle-related risk factors for AD outlined by Livingston et al., there are other factors that can impact AD risk and the PLR. Additionally, some of the AD risk factors have a comorbidity with one another, which could confound potential relationships between the individual risk factors, AD, and the PLR. Some of the prevalent confounding factors will be outlined in this section.

#### 4.3.1. Changes to the eye and AD

When assessing the PLR, it is evident that changes to the eye itself may impact the response, including retinal changes. There are many studies that have related vision changes to AD, as the eye is closely related to the brain – the retina shares important pathways, both structural and pathogenic, with the central nervous system (Cabrera DeBuc et al., 2018). AD may impact visual function early in the disease progression, and losses in visual function correlate with cognitive losses (Valenti, 2010). Rogers and Langa found that generally, poor vision that is left untreated is associated with cognitive decline and AD (Rogers and Langa, 2010).

There are also associations when considering, more specifically, changes to the retina and AD. Amyloid-beta and phosphorylated tau can accumulate in the retinas in early-stage cases of AD, which could be used as an early biomarker for AD (Koronyo et al., 2017; Gupta et al., 2021). The retinal nerve fiber layer thickness has been found to be smaller among AD patients when compared to healthy controls (Kirbas et al., 2013; Asanad et al., 2019; Gupta et al., 2021), as has the retinal ganglion cell layer, inner nuclear layer, and outer nuclear layer (Asanad et al., 2019). Cabrera DeBuc et al. suggest that retinal geometric vascular and functional parameters could be associated with retinal changes due to cognitive decline and could serve as a useful clinical marker of cognitive decline (Cabrera DeBuc et al., 2018).

It may be difficult to separate these changes in the eye associated with AD, from independent changes to the parasympathetic or sympathetic response resulting in an impaired PLR. It could be claimed that retinal degeneration and other retinal and optic nerve changes in AD could be, at least partially, responsible for the reduced PLR observed in AD; however, this is not supported by clinical observations of AD patients in a neuro-ophthalmological examination (Chang et al., 2014). Further research should be done to separate ocular and retinal changes to the PLR from direct AD-related changes.

#### 4.3.2. Population demographics and PLR

There are several demographics of a population that can influence the PLR. The specific factors that will be highlighted are age, sex, and living environments.

##### 4.3.2.1. Age

There are pupillary changes that occur with age in otherwise healthy adults. Many studies have shown that after growing until adolescence, the size of the pupil decreases with age (Feinberg et al., 1965; Winn et al., 1994; Bitsios et al., 1996; Fotiou et al., 2007a; Sharma et al., 2016; Eckstein et al., 2017). In terms of the PLR, Sharma et al. found that the amplitude of the PLR to blue light was reduced with age (Sharma et al., 2016). Bitsios et al., using green light, and Fotiou et al., using white light, similarly found a reduction, among older subjects, in the PLR, but did not find a difference in the latency (Bitsios et al., 1996; Fotiou et al., 2007a). This contradicts Feinberg and Podolak’s conclusion that pupillary latency increases with age (Feinberg et al., 1965). Although there are disputes as to whether pupillary latency changes with age, the finding that pupil size decreases with age is an important factor to consider when assessing the amplitudes of the PLR.

##### 4.3.2.2. Sex and gender

Sex and gender differences have been identified in AD prevalence, clinical manifestation, and prognosis (Kim et al., 2015). Women have a higher lifetime risk of developing AD compared to men (Farrer et al., 1997; Seshadri et al., 1997), however men have a shorter lifespan after diagnosis (Podcasy and Epperson, 2016).

Fan and Yao found that females have a higher parasympathetic activity and lower sympathetic activity when compared to males, consistent with findings presented in other cardiovascular studies (Fan and Yao, 2011). Van Stavern et al. found that there is a potential effect from an individual’s sex that could influence the PLR – an example provided is that males with a biomarker showed a reduced constriction percentage when compared to males without biomarkers, although it was not found to be statistically significant (Van Stavern et al., 2019). Further studies should be conducted to assess sex and gender difference in AD risk factors and the PLR, to draw adequate conclusions.

##### 4.3.2.3. Living environment

An individual’s living environment can also affect AD risk, in addition to the PLR. Livingston et al. identified air pollution as a risk factor for AD, likely due to vascular mechanisms (Livingston et al., 2020). Carey et al. investigated both air and noise pollution and found that higher levels of air and noise pollution correspond with higher risks of dementia (Carey et al., 2018). Interestingly, Paciência et al. found that the walkability of school neighborhoods was negatively associated with the pupillary response, specifically with the pupil constriction amplitude and redilation time, which they suggest is due to the school environments affecting the lung function of students, an effect which may be partially mediated by the autonomic nervous system (Paciência et al., 2019). As shown, there are several aspects of an individual’s living environment that can increase risk for AD and can impact the health of residents, however more research should be done to assess other living environment parameters with AD risk and the pupillary light response, while controlling for other factors.

#### 4.3.3. Comorbidity between Alzheimer’s disease risk factors

This review aimed to distinguish individual risk factors and their specific contributions, if any, to an impaired PLR. However, many of these risk factors have comorbidities, and as such it can be difficult to distinguish individual impacts. Some of the prevalent comorbidities will be discussed here.

There are several diseases and conditions that share risk factors with dementia, and conditions that may be side effects of dementia. Those living with dementia may not remember to tell family members or health professionals their symptoms, or may struggle to follow health and nutrition plans, which could increase infections (Livingston et al., 2020). A reverse causation between dementia and depression can also exist, where depressive symptoms may result from dementia neuropathology (Livingston et al., 2020). Prodromal dementia may also stop people from exercising, and so physical inactivity, like depression, may either be a consequence or cause of dementia (Livingston et al., 2020). Additionally, many of the risk factors of dementia are also risk factors for cardiovascular diseases including hypertension, obesity, and diabetes (Kivipelto et al., 2006), all of which are individual risk factors for the disease itself and have been shown, to varying extents, to have some impact on the PLR.

In terms of the relationship between risk factors, there are many. Several studies suggest that hearing impairment is associated with psychosocial problems, including depression or loneliness (Strawbridge et al., 2000; Nachtegaal et al., 2009; Pronk et al., 2011; Wang et al., 2016). Considering that depression and social isolation are additional risk factors for AD, this indicates a significant comorbidity. The ability to communicate with people depends significantly on hearing ability, and so hearing impairment can have a significant impact on social life, leading to social isolation and then, subsequently, to depression, cognitive decline, and dementia (Bowl and Dawson, 2019; Ralli et al., 2019). Further, the prevalence of hearing impairment is more common in diabetics than in non-diabetics, and Bainbridge et al. suggest that hearing impairment may be an under-recognized complication of diabetes (Bainbridge et al., 2008).

Diabetes and obesity are closely linked. Both conditions have a pathophysiology that is attributed to insulin resistance and insulin deficiency (Verma and Hussain, 2017). Obesity is associated with an increased risk of diabetes and is also associated with other health conditions including high blood pressure, high cholesterol levels, arthritis, and asthma (Mokdad et al., 2003). Physical activity is shown to be an important method of combating both diabetes and obesity, which further relates these risk factors (Verma and Hussain, 2017). Additionally, Mokdad et al. found that adults with less than a high school education had the highest rate of diabetes among all educational levels (Mokdad et al., 2003).

Education, understandably, can inform an individual’s social and lifestyle habits. Helliwell and Putnam state that education is usually the most important predictor of social engagement (Helliwell and Putnam, 1999). It is thus important to consider how education may be related to risk factors related to social engagement and, by extension, depression.

## 5. Concluding remarks

AD is rapidly increasing around the world, and the current aim is to prevent the onset of the disease. The pupillary light response has been shown to be impaired in current AD subjects, but it is currently unknown if it could be used as a tool in at-risk groups to predict AD risk. This review outlined prevalent AD risk factors and assessed the pupillary light responses evoked in AD subjects and those belonging to AD risk factor groups.

Traumatic brain injury, ocular and intracranial hypertension, alcohol consumption, depression, and diabetes are all AD risk factors that have demonstrated changes in the PLR, in varying time frames. Hearing loss, smoking, and genetic factors have had associations with changes to the parasympathetic activity, which could indicate an impaired PLR, however further research should be done to confirm this hypothesis. Genetic risk factors have additionally had limited direct associations with the PLR, and more research should be done to investigate this relationship. No conclusions could be drawn between level of education, social isolation, obesity, and physical inactivity and the PLR, mainly due to a lack of literature, so more research should be done to investigate any potential relationships. Further, there is a comorbidity between some AD risk factors, therefore further research is necessary to separate the individual impacts of these risk factors on the PLR.

With these findings, the PLR has clearly been shown to be impaired in current AD patients and in certain at-risk groups. Currently, pupillometry using the PLR has value as a confirmatory measure of AD. In the future, the PLR has the potential to be used as an early diagnostic tool for AD and could be used, in conjunction with a lifestyle, life-event, genetics, and physiological assessment, to identify when preventative measures should be taken for AD. To enable this, future research in this area should consider the impacts of individual lifestyle, genetic, life-event and physiological risk factors for AD and how these relate, if at all, to the PLR. Further, these future studies should ensure that a standard methodology for pupillometry measurements, as was proposed by Kelbsch et al. (2019), should be used to ensure that these studies can be easily compared.

## Author contributions

SS and DB contributed to the conception and initial design of the manuscript. SS performed the search and selection of the relevant studies and wrote the manuscript. JP, GH, MS, DB, and SS contributed to the manuscript revision and editing. All authors contributed to the article and approved the submitted version.

## Funding

This work was supported by Engineering and Physical Sciences Research Council UK through grant EP/S021507/1. SS was supported by the Rhodes Trust, GH was supported by Clarendon.

## Conflict of interest

The authors declare that the research was conducted in the absence of any commercial or financial relationships that could be construed as a potential conflict of interest.

## Publisher’s note

All claims expressed in this article are solely those of the authors and do not necessarily represent those of their affiliated organizations, or those of the publisher, the editors and the reviewers. Any product that may be evaluated in this article, or claim that may be made by its manufacturer, is not guaranteed or endorsed by the publisher.

## References

[ref1] Aguilar M. Stiles W. S. (1954). Saturation of the rod mechanism of the retina at high levels of stimulation. Optic. Acta 1, 59–65. doi: 10.1080/713818657

[ref2] Albanese E. Launer L. J. Egger M. Prince M. J. Giannakopoulos P. Wolters F. J.. (2017). Body mass index in midlife and dementia: systematic review and meta-regression analysis of 589,649 men and women followed in longitudinal studies. Alzheimer’s Dement. 8, 165–178. doi: 10.1016/j.dadm.2017.05.007, PMID: 28761927 PMC5520956

[ref3] Alber J. Goldfarb D. Thompson L. I. Arthur E. Hernandez K. Cheng D.. (2020). Developing retinal biomarkers for the earliest stages of Alzheimer’s disease: what we know, what we don’t, and how to move forward. Alzheimers Dement. 16, 229–243. doi: 10.1002/alz.12006, PMID: 31914225

[ref4] Amieva H. Ouvrard C. Giulioli C. Meillon C. Rullier L. Dartigues J. F. (2015). Self-reported hearing loss, hearing aids, and cognitive decline in elderly adults: a 25-year study. J. Am. Geriatr. Soc. 63, 2099–2104. doi: 10.1111/jgs.13649, PMID: 26480972

[ref5] Anderson E. A. Sinkey C. A. Lawton W. J. Mark A. L. (1989). Elevated sympathetic nerve activity in borderline hypertensive humans. Evidence from direct intraneural recordings. Hypertension 14, 177–183. doi: 10.1161/01.HYP.14.2.177, PMID: 2759678

[ref6] Anstey K. J. von Sanden C. Salim A. O'Kearney R. (2007). Smoking as a risk factor for dementia and cognitive decline: a meta-analysis of prospective studies. Am. J. Epidemiol. 166, 367–378. doi: 10.1093/aje/kwm116, PMID: 17573335

[ref7] Areosa Sastre A. Vernooij R. W. González-Colaço Harmand M. Martínez G. (2017). Effect of the treatment of type 2 diabetes mellitus on the development of cognitive impairment and dementia. Cochrane Database Syst. Rev. 6:CD003804. doi: 10.1002/14651858.CD003804.pub2, PMID: 28617932 PMC6481422

[ref8] Asanad S. Ross-Cisneros F. N. Nassisi M. Barron E. Karanjia R. Sadun A. A. (2019). The retina in Alzheimer’s disease: histomorphometric analysis of an ophthalmologic biomarker. Invest. Ophthalmol. Vis. Sci. 60, 1491–1500. doi: 10.1167/iovs.18-25966, PMID: 30973577 PMC6892387

[ref9] Ashok A. Singh N. Chaudhary S. Bellamkonda V. Kritikos A. E. Wise A. S.. (2020). Retinal Degeneration and Alzheimer’s Disease: An Evolving Link. Int. J. Mol. Sci. 21:7290. doi: 10.3390/ijms21197290, PMID: 33023198 PMC7582766

[ref10] Association, A (2019). 2019 Alzheimer’s disease facts and figures. Alzheimers Dement. 15, 321–387. doi: 10.1016/j.jalz.2019.01.010

[ref11] Babulal G. M. Quiroz Y. T. Albensi B. C. Arenaza-Urquijo E. Astell A. J. Babiloni C.. (2019). Perspectives on ethnic and racial disparities in Alzheimer’s disease and related dementias: update and areas of immediate need. Alzheimers Dement. 15, 292–312. doi: 10.1016/j.jalz.2018.09.009, PMID: 30555031 PMC6368893

[ref12] Baglietto-Vargas D. Shi J. Yaeger D. M. Ager R. LaFerla F. M. (2016). Diabetes and Alzheimer’s disease crosstalk. Neurosci. Biobehav. Rev. 64, 272–287. doi: 10.1016/j.neubiorev.2016.03.005 26969101

[ref13] Bainbridge K. E. Hoffman H. J. Cowie C. C. (2008). Diabetes and hearing impairment in the United States: audiometric evidence from the National Health and nutrition examination survey, 1999 to 2004. Ann. Intern. Med. 149, 1–10. doi: 10.7326/0003-4819-149-1-200807010-00231 18559825 PMC2803029

[ref14] Baker M. L. Marino Larsen E. K. Kuller L. H. Klein R. Klein B. E. K. Siscovick D. S.. (2007). Retinal microvascular signs, cognitive function, and dementia in older persons. Stroke 38, 2041–2047. doi: 10.1161/STROKEAHA.107.483586, PMID: 17525385

[ref15] Bär K.-J. Greiner W. Jochum T. Friedrich M. Wagner G. Sauer H. (2004). The influence of major depression and its treatment on heart rate variability and pupillary light reflex parameters. J. Affect. Disord. 82, 245–252. doi: 10.1016/j.jad.2003.12.016, PMID: 15488253

[ref16] Barnes D. E. Byers A. L. Gardner R. C. Seal K. H. Boscardin W. J. Yaffe K. (2018). Association of Mild Traumatic Brain Injury with and without Loss of consciousness with dementia in US military veterans. JAMA Neurol. 75, 1055–1061. doi: 10.1001/jamaneurol.2018.0815, PMID: 29801145 PMC6143113

[ref17] Baum P. Petroff D. Classen J. Kiess W. Blüher S. (2013). Dysfunction of autonomic nervous system in childhood obesity: a cross-sectional study. PLoS One 8:e54546. doi: 10.1371/journal.pone.0054546, PMID: 23358101 PMC3554723

[ref18] Beltrán J. García-Vázquez M. S. Benois-Pineau J. Gutierrez-Robledo L. M. Dartigues J. F. (2018). Computational techniques for eye movements analysis towards supporting early diagnosis of Alzheimer’s disease: a review. Comp. Math. Methods Med. 2018:e2676409. doi: 10.1155/2018/2676409, PMID: 29887912 PMC5985110

[ref19] Berman G. Muttuvelu D. Berman D. Larsen J. I. Licht R. W. Ledolter J.. (2018). Decreased retinal sensitivity in depressive disorder: a controlled study. Acta Psychiatr. Scand. 137, 231–240. doi: 10.1111/acps.12851, PMID: 29336011

[ref20] Bista Karki S. Coppell K. J. Mitchell L. V. Ogbuehi K. C. (2020). Dynamic Pupillometry in type 2 diabetes: pupillary autonomic dysfunction and the severity of diabetic retinopathy. Clin. Ophthalmol. 14, 3923–3930. doi: 10.2147/OPTH.S279872, PMID: 33244218 PMC7683350

[ref21] Bitsios P. Prettyman R. Szabadi E. (1996). Changes in autonomic function with age: a study of pupillary kinetics in healthy young and old people. Age Ageing 25, 432–438. doi: 10.1093/ageing/25.6.432 9003878

[ref22] Blüher S. Petroff D. Keller A. Wagner A. Classen J. Baum P. (2015). Effect of a 1-year obesity intervention (KLAKS program) on preexisting autonomic nervous dysfunction in childhood obesity. J. Child Neurol. 30, 1174–1181. doi: 10.1177/0883073814555190, PMID: 25406153

[ref23] Boucart M. Bubbico G. Szaffarczyk S. Pasquier F. (2014). Animal spotting in Alzheimer’s disease: an eye tracking study of object categorization. J. Alzheimer’s Dis. 39, 181–189. doi: 10.3233/JAD-131331, PMID: 24121969

[ref24] Bowl M. R. Dawson S. J. (2019). Age-related hearing loss. Cold Spring Harb. Perspect. Med. 9:a033217. doi: 10.1101/cshperspect.a033217 30291149 PMC6671929

[ref25] Cabrera DeBuc D. Somfai G. M. Arthur E. Kostic M. Oropesa S. Mendoza Santiesteban C. (2018). Investigating multimodal diagnostic eye biomarkers of cognitive impairment by measuring vascular and neurogenic changes in the retina. Front. Physiol. 9:1721. doi: 10.3389/fphys.2018.01721, PMID: 30574092 PMC6291749

[ref26] Caraci F. Copani A. Nicoletti F. Drago F. (2010). Depression and Alzheimer’s disease: neurobiological links and common pharmacological targets. Eur. J. Pharmacol. 626, 64–71. doi: 10.1016/j.ejphar.2009.10.022, PMID: 19837057

[ref27] Carey I. M. Anderson H. R. Atkinson R. W. Beevers S. D. Cook D. G. Strachan D. P.. (2018). Are noise and air pollution related to the incidence of dementia? A cohort study in London, England. BMJ Open 8:e022404. doi: 10.1136/bmjopen-2018-022404, PMID: 30206085 PMC6144407

[ref28] Cataldo J. K. Prochaska J. J. Glantz S. A. (2010). Cigarette smoking is a risk factor for Alzheimer’s disease: an analysis controlling for tobacco industry affiliation. J. Alzheimer’s Dis. 19, 465–480. doi: 10.3233/JAD-2010-1240 20110594 PMC2906761

[ref29] Chang L. Y. L. Lowe J. Ardiles A. Lim J. Grey A. C. Robertson K.. (2014). Alzheimer’s disease in the human eye. Clinical tests that identify ocular and visual information processing deficit as biomarkers. Alzheimers Dement. 10, 251–261. doi: 10.1016/j.jalz.2013.06.004, PMID: 24011928

[ref30] Chang C.-C. H. Zhao Y. Lee C. W. Ganguli M. (2012). Smoking, death, and Alzheimer disease: a case of competing risks. Alzheimer Dis. Assoc. Disord. 26, 300–306. doi: 10.1097/wad.0b013e3182420b6e, PMID: 22185783 PMC3321062

[ref31] Chen J. W. Gombart Z. J. Rogers S. Gardiner S. K. Cecil S. Bullock R. M. (2011). Pupillary reactivity as an early indicator of increased intracranial pressure: the introduction of the neurological pupil index. Surg. Neurol. Int. 2:82. doi: 10.4103/2152-7806.82248, PMID: 21748035 PMC3130361

[ref32] Cheung C. Y. Ikram M. K. Chen C. Wong T. Y. (2017). Imaging retina to study dementia and stroke. Prog. Retin. Eye Res. 57, 89–107. doi: 10.1016/j.preteyeres.2017.01.001, PMID: 28057562

[ref33] Chi S. Yu J. T. Tan M. S. Tan L. (2014). Depression in Alzheimer’s disease: epidemiology, mechanisms, and management. J. Alzheimers Dis. 42, 739–755. doi: 10.3233/JAD-140324, PMID: 24946876

[ref34] Chiasseu M. Alarcon-Martinez L. Belforte N. Quintero H. Dotigny F. Destroismaisons L.. (2017). Tau accumulation in the retina promotes early neuronal dysfunction and precedes brain pathology in a mouse model of Alzheimer’s disease. Mol. Neurodegener. 12:58. doi: 10.1186/s13024-017-0199-3, PMID: 28774322 PMC5543446

[ref35] Chida K. Takasu T. Kawamura H. (1998). Changes in sympathetic and parasympathetic function in alcoholic neuropathy. Japan. J. Alcohol Stud. Drug Depend. 33, 45–55. PMID: 9549310

[ref36] Choi D. Choi S. Park S. M. (2018). Effect of smoking cessation on the risk of dementia: a longitudinal study. Ann. Clin. Transl. Neurol. 5, 1192–1199. doi: 10.1002/acn3.633 30349854 PMC6186929

[ref37] Chougule P. S. Najjar R. P. Finkelstein M. T. Kandiah N. Milea D. (2019). Light-induced pupillary responses in Alzheimer’s disease. Front. Neurol. 10:360. doi: 10.3389/fneur.2019.00360, PMID: 31031692 PMC6473037

[ref38] de la Rosa A. Olaso-Gonzalez G. Arc-Chagnaud C. Millan F. Salvador-Pascual A. García-Lucerga C.. (2020). Physical exercise in the prevention and treatment of Alzheimer’s disease. J. Sport Health Sci. 9, 394–404. doi: 10.1016/j.jshs.2020.01.004, PMID: 32780691 PMC7498620

[ref39] de Winter J. Petermeijer S. Kooijman L. Dodou D. (2021). Replicating five pupillometry studies of Eckhard Hess. 10.1016/j.ijpsycho.2021.03.003 33766646

[ref40] Deal J. A. Betz J. Yaffe K. Harris T. Purchase-Helzner E. Satterfield S.. (2017). Hearing impairment and incident dementia and cognitive decline in older adults: the health ABC study. J. Gerontol. A Biol. Sci. Med. Sci. 72, glw069–glw709. doi: 10.1093/gerona/glw069, PMID: 27071780 PMC5964742

[ref41] Deal J. A. Sharrett A. R. Albert M. S. Coresh J. Mosley T. H. Knopman D.. (2015). Hearing impairment and cognitive decline: a pilot study conducted within the atherosclerosis risk in communities neurocognitive study. Am. J. Epidemiol. 181, 680–690. doi: 10.1093/aje/kwu333, PMID: 25841870 PMC4408947

[ref42] Debanne S. M. Bielefeld R. A. Cheruvu V. K. Fritsch T. Rowland D. Y. (2007). Alzheimer’s disease and smoking: bias in cohort studies. J. Alzheimer’s Dis. 11, 313–321. doi: 10.3233/jad-2007-11308, PMID: 17851182

[ref43] den Haan J. Morrema T. H. J. Verbraak F. D. de Boer J. F. Scheltens P. Rozemuller A. J.. (2018). Amyloid-beta and phosphorylated tau in post-mortem Alzheimer's disease retinas. Acta Neuropathol. Commun. 6:147. doi: 10.1186/s40478-018-0650-x, PMID: 30593285 PMC6309096

[ref44] den Haan J. Verbraak F. D. Visser P. J. Bouwman F. H. (2017). Retinal thickness in Alzheimer’s disease: a systematic review and meta-analysis. Alzheimer’s Dement. 6, 162–170. doi: 10.1016/j.dadm.2016.12.014, PMID: 28275698 PMC5328759

[ref45] Dhingra D. Kaur S. Ram J. (2019). Illicit drugs: effects on eye. Indian J. Med. Res. 150, 228–238. doi: 10.4103/ijmr.IJMR_1210_17 31719293 PMC6886135

[ref46] Ding J. Davis-Plourde K. L. Sedaghat S. Tully P. J. Wang W. Phillips C.. (2020). Antihypertensive medications and risk for incident dementia and Alzheimer’s disease: a meta-analysis of individual participant data from prospective cohort studies. Lancet Neurol. 19, 61–70. doi: 10.1016/S1474-4422(19)30393-X, PMID: 31706889 PMC7391421

[ref47] Dotson V. M. Beydoun M. A. Zonderman A. B. (2010). Recurrent depressive symptoms and the incidence of dementia and mild cognitive impairment. Neurology 75, 27–34. doi: 10.1212/WNL.0b013e3181e62124 20603482 PMC2906403

[ref48] Eckstein M. K. Guerra-Carrillo B. Miller Singley A. T. Bunge S. A. (2017). Beyond eye gaze: what else can eyetracking reveal about cognition and cognitive development? Dev. Cogn. Neurosci. 25, 69–91. doi: 10.1016/j.dcn.2016.11.001, PMID: 27908561 PMC6987826

[ref49] Ellis C. J. (1981). The pupillary light reflex in normal subjects. Br. J. Ophthalmol. 65, 754–759. doi: 10.1136/bjo.65.11.754, PMID: 7326222 PMC1039657

[ref50] Fan X. Yao G. (2011). Modeling transient pupillary light reflex induced by a short light flash. IEEE Trans. Biomed. Eng. 58, 36–42. doi: 10.1109/TBME.2010.2080678 20876003 PMC4318650

[ref51] Fann J. R. Ribe A. R. Pedersen H. S. Fenger-Grøn M. Christensen J. Benros M. E.. (2018). Long-term risk of dementia among people with traumatic brain injury in Denmark: a population-based observational cohort study. Lancet Psychiatry 5, 424–431. doi: 10.1016/S2215-0366(18)30065-8, PMID: 29653873

[ref52] Faraco G. Iadecola C. (2013). Hypertension. Hypertension 62, 810–817. doi: 10.1161/HYPERTENSIONAHA.113.01063, PMID: 23980072 PMC3847558

[ref53] Farrer L. A. Cupples L. A. Haines J. L. Hyman B. Kukull W. A. Mayeux R.. (1997). Effects of age, sex, and ethnicity on the association between apolipoprotein E genotype and Alzheimer disease: a meta-analysis. JAMA 278, 1349–1356. doi: 10.1001/jama.1997.03550160069041 9343467

[ref54] Feigl B. Ojha G. Hides L. Zele A. J. (2018). Melanopsin-driven pupil response and light exposure in non-seasonal major depressive disorder. Front. Neurol. 9:764. doi: 10.3389/fneur.2018.00764, PMID: 30271376 PMC6146094

[ref55] Feigl B. Zele A. J. Fader S. M. Howes A. N. Hughes C. E. Jones K. A.. (2012). The post-illumination pupil response of melanopsin-expressing intrinsically photosensitive retinal ganglion cells in diabetes. Acta Ophthalmol. 90, e230–e234. doi: 10.1111/j.1755-3768.2011.02226.x, PMID: 21883986

[ref56] Feil M. Moser B. Abegg M. (2017). The interaction of pupil response with the vergence system. Graefe’s Arch. Clin. Exp. Ophthalmol. 255, 2247–2253. doi: 10.1007/s00417-017-3770-2 28815298

[ref57] Feinberg R. Podolak E. Georgetown Clinical Research Institute. (1965). Latency of pupillary reflex to light stimulation and its relationship to aging. AM 65-25. Available at: https://rosap.ntl.bts.gov/view/dot/20753 [Accessed December 21, 2021].

[ref58] Fernández G. Manes F. Politi L. E. Orozco D. Schumacher M. Castro L.. (2016). Patients with mild Alzheimer’s disease fail when using their working memory: evidence from the eye tracking technique. J. Alzheimers Dis. 50, 827–838. doi: 10.3233/JAD-150265, PMID: 26836011

[ref59] Ferrari G. L. Marques J. L. B. Gandhi R. A. Emery C. J. Tesfaye S. Heller S. R.. (2007). ‘An approach to the assessment of diabetic neuropathy based on dynamic Pupillometry’, In 2007 29th Annual International Conference of the IEEE Engineering in Medicine and Biology Society. 2007 29th Annual International Conference of the IEEE Engineering in Medicine and Biology Society, pp. 557–560. 10.1109/IEMBS.2007.4352351 18002017

[ref60] Ferrario E. Molaschi M. Villa L. Varetto O. Bogetto C. Nuzzi R. (1998). Is videopupillography useful in the diagnosis of Alzheimer’s disease? Neurology 50, 642–644. doi: 10.1212/WNL.50.3.642, PMID: 9521249

[ref62] Fotiou D. F. Brozou C. G. Haidich A.-B. Tsiptsios D. Nakou M. Kabitsi A.. (2007b). Pupil reaction to light in Alzheimer’s disease: evaluation of pupil size changes and mobility. Aging Clin. Exp. Res. 19, 364–371. doi: 10.1007/BF03324716, PMID: 18007114

[ref63] Fotiou D. F. Brozou C. G. Tsiptsios D. J. Fotiou A. Kabitsi A. Nakou M.. (2007a). Effect of age on pupillary light reflex: evaluation of pupil mobility for clinical practice and research. Electromyogr. Clin. Neurophysiol. 47, 11–22. PMID: 17375877

[ref64] Fotiou F. Fountoulakis K. N. Tsolaki M. Goulas A. Palikaras A. (2000). Changes in pupil reaction to light in Alzheimer’s disease patients: a preliminary report. Int. J. Psychophysiol. 37, 111–120. doi: 10.1016/S0167-8760(00)00099-4, PMID: 10828379

[ref65] Fotiou D. Kaltsatou A. Tsiptsios D. Nakou M. (2015). Evaluation of the cholinergic hypothesis in Alzheimer’s disease with neuropsychological methods. Aging Clin. Exp. Res. 27, 727–733. doi: 10.1007/s40520-015-0321-8, PMID: 25749905

[ref66] Fotiou F. Fountoulakis K. N. Goulas A. Alexopoulos L. Palikaras A. (2008). Automated standardized pupillometry with optical method for purposes of clinical practice and research. Available at: https://onlinelibrary.wiley.com/doi/full/10.1046/j.1365-2281.2000.00259.x?casa_token=9ERgTtNnHScAAAAA%3A8MKl4grzRu1wP_tc2TzqYjLCs38cjPYL3fkn8VcJzUoVrjtwa_9KAYk6nPZnGf_bmuuZ7_jPi9g-iQ [Accessed February 8, 2022]. 10.1046/j.1365-2281.2000.00259.x 10971544

[ref67] Fountoulakis K. Fotiou F. Iacovides A. Tsiptsios J. Goulas A. Tsolaki M.. (1999). Changes in pupil reaction to light in melancholic patients. Int. J. Psychophysiol. 31, 121–128. doi: 10.1016/s0167-8760(98)00046-4, PMID: 9987058

[ref68] Fritze T. Teipel S. Óvári A. Kilimann I. Witt G. Doblhammer G. (2016). Hearing impairment affects dementia incidence. An analysis based on longitudinal health claims data in Germany. PLoS One 11:e0156876. doi: 10.1371/journal.pone.0156876, PMID: 27391486 PMC4938406

[ref69] Frost S. Kanagasingam Y. Sohrabi H. Bourgeat P. Villemagne V. Rowe C. C.. (2013). Pupil response biomarkers for early detection and monitoring of Alzheimer’s disease. Curr. Alzheimer Res. 10, 931–939. doi: 10.2174/15672050113106660163, PMID: 24117119

[ref70] Frost S. M. Kanagasingam Y. Sohrabi H. Taddei K. Bateman R. Morris J.. (2013). Pupil response biomarkers distinguish amyloid precursor protein mutation carriers from non-carriers. Curr. Alzheimer Res. 10, 790–796. doi: 10.2174/15672050113109990154, PMID: 23919771 PMC3879087

[ref71] Frost S. Robinson L. Rowe C. C. Ames D. Masters C. L. Taddei K.. (2017). Evaluation of cholinergic deficiency in preclinical Alzheimer’s disease using Pupillometry. J. Ophthalmol. 2017:e7935406. doi: 10.1155/2017/7935406, PMID: 28894607 PMC5574262

[ref72] Gallacher J. Ilubaera V. Ben-Shlomo Y. Bayer A. Fish M. Babisch W.. (2012). Auditory threshold, phonologic demand, and incident dementia. Neurology 79, 1583–1590. doi: 10.1212/WNL.0b013e31826e263d, PMID: 23019269

[ref73] Granholm E. Morris S. Galasko D. Shults C. Rogers E. Vukov B. (2003). Tropicamide effects on pupil size and pupillary light reflexes in Alzheimer’s and Parkinson’s disease. Int. J. Psychophysiol. 47, 95–115. doi: 10.1016/s0167-8760(02)00122-8, PMID: 12568941

[ref74] Granholm E. L. Panizzon M. S. Elman J. A. Jak A. J. Hauger R. L. Bondi M. W.. (2017). Pupillary responses as a biomarker of&nbsp;early risk for Alzheimer’s disease. J. Alzheimers Dis. 56, 1419–1428. doi: 10.3233/JAD-161078, PMID: 28157098 PMC5808562

[ref75] Granholm E. Steinhauer S. R. (2004). Pupillometric measures of cognitive and emotional processes. Int. J. Psychophysiol. 52, 1–6. doi: 10.1016/j.ijpsycho.2003.12.001, PMID: 15003368

[ref76] Griffiths T. D. Lad M. Kumar S. Holmes E. McMurray B. Maguire E. A.. (2020). How can hearing loss cause dementia? Neuron 108, 401–412. doi: 10.1016/j.neuron.2020.08.003, PMID: 32871106 PMC7664986

[ref77] Grozdanic S. D. Kwon Y. H. Sakaguchi D. S. Kardon R. H. Sonea I. M. (2004). Functional evaluation of retina and optic nerve in the rat model of chronic ocular hypertension. Exp. Eye Res. 79, 75–83. doi: 10.1016/j.exer.2004.02.011, PMID: 15183102

[ref78] Grozdanic S. D. Sakaguchi D. S. Kwon Y. H. Kardon R. H. Sonea I. M. (2003). Functional characterization of retina and optic nerve after acute ocular ischemia in rats. Invest. Ophthalmol. Vis. Sci. 44, 2597–2605. doi: 10.1167/iovs.02-0600, PMID: 12766062

[ref79] Gupta V. B. Chitranshi N. den Haan J. Mirzaei M. You Y. Lim J. K. H.. (2021). Retinal changes in Alzheimer’s disease— integrated prospects of imaging, functional and molecular advances. Prog. Retin. Eye Res. 82:100899. doi: 10.1016/j.preteyeres.2020.100899, PMID: 32890742

[ref80] Gurgel R. K. Ward P. D. Schwartz S. Norton M. C. Foster N. L. Tschanz J. A. T. (2014). Relationship of hearing loss and dementia: a prospective, population-based study. Otol. Neurotol. 35, 775–781. doi: 10.1097/MAO.0000000000000313, PMID: 24662628 PMC4024067

[ref81] Hall C. A. Chilcott R. P. (2018). Eyeing up the future of the pupillary light reflex in neurodiagnostics. Diagnostics 8:19. doi: 10.3390/diagnostics8010019, PMID: 29534018 PMC5872002

[ref82] Hanna S. T. (2006). Nicotine effect on cardiovascular system and ion channels. J. Cardiovasc. Pharmacol. 47, 348–358. doi: 10.1097/01.fjc.0000205984.13395.9e 16633075

[ref83] Hart de Ruyter F. J. Morrema T. H. J. den Haan J. Netherlands Brain Bank Twisk J. W. R. de Boer J. F.. (2023). Phosphorylated tau in the retina correlates with tau pathology in the brain in Alzheimer’s disease and primary tauopathies. Acta Neuropathol. 145, 197–218. doi: 10.1007/s00401-022-02525-1, PMID: 36480077

[ref84] Hasson D. Theorell T. Liljeholm-Johansson Y. Canlon B. (2009). Psychosocial and physiological correlates of self-reported hearing problems in male and female musicians in symphony orchestras. Int. J. Psychophysiol. 74, 93–100. doi: 10.1016/j.ijpsycho.2009.07.009, PMID: 19666059

[ref85] Hayes G. Pinto J. Sparks S. N. Wang C. Suri S. Bulte D. P. (2022). Vascular smooth muscle cell dysfunction in neurodegeneration. Front. Neurosci. 16:1010164. doi: 10.3389/fnins.2022.1010164, PMID: 36440263 PMC9684644

[ref86] Helliwell J.F. Putnam R.D. (1999) ‘Education and social capital’. National Bureau of Economic Research (Working Paper Series).

[ref87] Hersi M. Irvine B. Gupta P. Gomes J. Birkett N. Krewski D. (2017). Risk factors associated with the onset and progression of Alzheimer’s disease: a systematic review of the evidence. Neurotoxicology 61, 143–187. doi: 10.1016/j.neuro.2017.03.006, PMID: 28363508

[ref88] Hess E. H. (1965). Attitude and pupil size. Sci. Am. 212, 46–54. doi: 10.1038/scientificamerican0465-46 14261525

[ref89] Hess E.H. Polt J.M. (1960) ‘Pupil size as related to interest value of visual stimuli’, Science. doi: 10.1126/science.132.3423.349 [Preprint]. 14401489

[ref90] Higuchi S. Matsushita S. Hasegawa Y. Muramatsu T. Arai H. Hayashida M. (1997). Apolipoprotein E epsilon 4 allele and pupillary response to tropicamide. Am. J. Psychiatry 154, 694–696. doi: 10.1176/ajp.154.5.694, PMID: 9137131

[ref91] Hreidarsson Á. B. Gundersen H. J. G. (1985). The pupillary response to light in type 1 (insulin-dependent) diabetes. Diabetologia 28, 815–821. doi: 10.1007/BF00291070, PMID: 4085694

[ref92] Ishibashi F. Kojima R. Taniguchi M. Kosaka A. Uetake H. Tavakoli M. (2017). The preferential impairment of pupil constriction stimulated by blue light in patients with type 2 diabetes without autonomic neuropathy. J. Diabetes Res. 2017, 2017:e6069730. doi: 10.1155/2017/6069730, PMID: 28421205 PMC5380853

[ref93] Jahns F.-P. Miroz J. P. Messerer M. Daniel R. T. Taccone F. S. Eckert P.. (2019). Quantitative pupillometry for the monitoring of intracranial hypertension in patients with severe traumatic brain injury. Crit. Care 23:155. doi: 10.1186/s13054-019-2436-3, PMID: 31046817 PMC6498599

[ref94] Jindou K. Yamasaki H. Yamamoto O. Nakano T. Yamamoto S. Yamada M. (2010). ‘An investigation on detection technique of Driver’s condition based on pupillary light reflex characteristics’, 대한전자공학회 기타 간행물, pp. 192–197.

[ref95] Johnson J. C. S. Marshall C. R. Weil R. S. Bamiou D. E. Hardy C. J. D. Warren J. D. (2021). Hearing and dementia: from ears to brain. Brain J. Neurol. 144, 391–401. doi: 10.1093/brain/awaa429, PMID: 33351095 PMC7940169

[ref96] Julius S. Krause L. Schork N. J. Mejia A. D. Jones K. A. van de Ven C.. (1991). Hyperkinetic borderline hypertension in Tecumseh, Michigan. J. Hypertens. 9, 77–84. doi: 10.1097/00004872-199101000-00012, PMID: 1848264

[ref97] Julius S. Pascual A. V. London R. (1971). Role of parasympathetic inhibition in the hyperkinetic type of borderline hypertension. Circulation 44, 413–418. doi: 10.1161/01.CIR.44.3.413, PMID: 5097443

[ref98] Kaifie A. Reugels M. Kraus T. Kursawe M. (2021). The pupillary light reflex (PLR) as a marker for the ability to work or drive – a feasibility study. J. Occup. Med. Toxicol. 16:39. doi: 10.1186/s12995-021-00330-2, PMID: 34493308 PMC8422642

[ref99] Karavanaki K. Davies A. G. Hunt L. P. Morgan M. H. Baum J. D. (1994). Pupil size in diabetes. Arch. Dis. Child. 71, 511–515. doi: 10.1136/adc.71.6.511, PMID: 7726610 PMC1030087

[ref100] Kawasaki A. Ouanes S. Crippa S. V. Popp J. (2020). Early-stage Alzheimer’s disease does not Alter pupil responses to colored light stimuli. J. Alzheimer’s Dis. 75, 1273–1282. doi: 10.3233/JAD-200120, PMID: 32417780

[ref101] Kelbsch C. Strasser T. Chen Y. Feigl B. Gamlin P. D. Kardon R.. (2019). Standards in Pupillography. Front. Neurol. 10:129. doi: 10.3389/fneur.2019.00129, PMID: 30853933 PMC6395400

[ref102] Kiely K. M. Gopinath B. Mitchell P. Luszcz M. Anstey K. J. (2012). Cognitive, health, and sociodemographic predictors of longitudinal decline in hearing acuity among older adults. J. Gerontol. A Biol. Sci. Med. Sci. 67, 997–1003. doi: 10.1093/gerona/gls066, PMID: 22415523

[ref103] Kim S. Kim M. J. Kim S. Kang H. S. Lim S. W. Myung W.. (2015). Gender differences in risk factors for transition from mild cognitive impairment to Alzheimer’s disease: a CREDOS study. Compr. Psychiatry 62, 114–122. doi: 10.1016/j.comppsych.2015.07.002, PMID: 26343475

[ref104] Kirbas S. Turkyilmaz K. Anlar O. Tufekci A. Durmus M. (2013). Retinal nerve Fiber layer thickness in patients with Alzheimer disease. J. Neuroophthalmol. 33, 58–61. doi: 10.1097/WNO.0b013e318267fd5f 22918296

[ref105] Kivipelto M. Ngandu T. Laatikainen T. Winblad B. Soininen H. Tuomilehto J. (2006). Risk score for the prediction of dementia risk in 20 years among middle aged people: a longitudinal, population-based study. Lancet Neurol. 5, 735–741. doi: 10.1016/S1474-4422(06)70537-3, PMID: 16914401

[ref106] Koronyo Y. Biggs D. Barron E. Boyer D. S. Pearlman J. A. Au W. J.. (2017). Retinal amyloid pathology and proof-of-concept imaging trial in Alzheimer's disease. JCI Insight 2:e93621. doi: 10.1172/jci.insight.93621, PMID: 28814675 PMC5621887

[ref107] Kramer S. E. Kapteyn T. S. Festen J. M. Kuik D. J. (1997). Assessing aspects of auditory handicap by means of pupil dilatation. Audiology 36, 155–164. doi: 10.3109/00206099709071969, PMID: 9193733

[ref108] Kremen W. S. Beck A. Elman J. A. Gustavson D. E. Reynolds C. A. Tu X. M.. (2019). Influence of young adult cognitive ability and additional education on later-life cognition. Proc. Natl. Acad. Sci. 116, 2021–2026. doi: 10.1073/pnas.1811537116, PMID: 30670647 PMC6369818

[ref109] Kurz A. Marquard R. Fremke S. Leipert K. (1997). Pupil dilation response to tropicamide: a biological test for Alzheimer’s disease? Pharmacopsychiatry 30, 12–15. doi: 10.1055/s-2007-979476 9065964

[ref110] Lane C. A. Barnes J. Nicholas J. M. Sudre C. H. Cash D. M. Parker T. D.. (2019). Associations between blood pressure across adulthood and late-life brain structure and pathology in the neuroscience substudy of the 1946 British birth cohort (insight 46): an epidemiological study. Lancet Neurol. 18, 942–952. doi: 10.1016/S1474-4422(19)30228-5, PMID: 31444142 PMC6744368

[ref111] Lanting P. Bos J. E. Aartsen J. Schuman L. Reichert-Thoen J. Heimans J. J. (1990). Assessment of pupillary light reflex latency and darkness adapted pupil size in control subjects and in diabetic patients with and without cardiovascular autonomic neuropathy. J. Neurol. Neurosurg. Psychiatry 53, 912–914. doi: 10.1136/jnnp.53.10.912, PMID: 2266375 PMC488257

[ref112] Lanting P. Strijers R. L. M. Bos J. E. Faes T. J. C. Heimans J. J. (1991). The cause of increased pupillary light reflex latencies in diabetic patients: the relationship between pupillary light reflex and visual evoked potential latencies. Electroencephalogr. Clin. Neurophysiol. 78, 111–115. doi: 10.1016/0013-4694(91)90110-p, PMID: 1704833

[ref113] Laurenzo S. A. Kardon R. Ledolter J. Poolman P. Schumacher A. M. Potash J. B.. (2016). Pupillary response abnormalities in depressive disorders. Psychiatry Res. 246, 492–499. doi: 10.1016/j.psychres.2016.10.039, PMID: 27821359 PMC5161673

[ref114] Lee A. J. Rochtchina E. Wang J. J. Healey P. R. Mitchell P. (2003). Does smoking affect intraocular pressure? Findings from the Blue Mountains eye study. J. Glaucoma 12, 209–212. doi: 10.1097/00061198-200306000-00005, PMID: 12782837

[ref115] Lerner A. G. Bernabé-Ortiz A. Ticse R. Hernandez A. Huaylinos Y. Pinto M. E.. (2015). Type 2 diabetes and cardiac autonomic neuropathy screening using dynamic pupillometry. Diab. Med. 32, 1470–1478. doi: 10.1111/dme.12752, PMID: 25761508 PMC4567976

[ref116] Lin F. R. Ferrucci L. Metter E. J. An Y. Zonderman A. B. Resnick S. M. (2011a). Hearing loss and cognition in the Baltimore longitudinal study of aging. Neuropsychology 25, 763–770. doi: 10.1037/a0024238, PMID: 21728425 PMC3193888

[ref117] Lin F. R. Metter E. J. O’Brien R. J. Resnick S. M. Zonderman A. B. Ferrucci L. (2011b). Hearing loss and incident dementia. Arch. Neurol. 68, 214–220. doi: 10.1001/archneurol.2010.362, PMID: 21320988 PMC3277836

[ref118] Liu C.-C. Kanekiyo T. Xu H. Bu G. (2013). Apolipoprotein E and Alzheimer disease: risk, mechanisms, and therapy. Nat. Rev. Neurol. 9, 106–118. doi: 10.1038/nrneurol.2012.263, PMID: 23296339 PMC3726719

[ref119] Liu Y. Liu F. Grundke-Iqbal I. Iqbal K. Gong C. X. (2009). Brain glucose transporters, O-GlcNAcylation and phosphorylation of tau in diabetes and Alzheimer’s disease. J. Neurochem. 111, 242–249. doi: 10.1111/j.1471-4159.2009.06320.x, PMID: 19659459 PMC2760012

[ref120] Livingston G. Huntley J. Sommerlad A. Ames D. Ballard C. Banerjee S.. (2020). Dementia prevention, intervention, and care: 2020 report of the lancet commission. Lancet 396, 413–446. doi: 10.1016/S0140-6736(20)30367-6, PMID: 32738937 PMC7392084

[ref121] Livingston G. Sommerlad A. Orgeta V. Costafreda S. G. Huntley J. Ames D.. (2017). Dementia prevention, intervention, and care. Lancet 390, 2673–2734. doi: 10.1016/S0140-6736(17)31363-6 28735855

[ref122] Loewenfeld I. E. (1958). Mechanisms of reflex dilatation of the pupil. Doc. Ophthalmol. 12, 185–448. doi: 10.1007/BF00913471 13609524

[ref123] London A. Benhar I. Schwartz M. (2013). The retina as a window to the brain—from eye research to CNS disorders. Nat. Rev. Neurol. 9, 44–53. doi: 10.1038/nrneurol.2012.227, PMID: 23165340

[ref124] Luchsinger J. A. Gustafson D. R. (2009). Adiposity and Alzheimer’s disease. Curr. Opin. Clin. Nutr. Metab. Care 12, 15–21. doi: 10.1097/MCO.0b013e32831c8c71, PMID: 19057182 PMC2771208

[ref125] Mackay D. F. Russell E. R. Stewart K. MacLean J. A. Pell J. P. Stewart W. (2019). Neurodegenerative disease mortality among former professional soccer players. N. Engl. J. Med. 381, 1801–1808. doi: 10.1056/NEJMoa1908483, PMID: 31633894 PMC8747032

[ref126] Mackersie C. L. MacPhee I. X. Heldt E. W. (2015). Effects of hearing loss on heart rate variability and skin conductance measured during sentence recognition in noise. Ear Hear. 36, 145–154. doi: 10.1097/AUD.0000000000000091, PMID: 25170782 PMC4272605

[ref127] Mancia G. Grassi G. (2014). The autonomic nervous system and hypertension. Circ. Res. 114, 1804–1814. doi: 10.1161/CIRCRESAHA.114.302524 24855203

[ref128] Mansouri K. Pajic B. Hafezi F. (2015). Effect of cigarette smoking on intraocular pressure. J Cataract Refract Surg 41, 682–683. doi: 10.1016/j.jcrs.2014.11.040, PMID: 25804587

[ref129] Mazziotti R. Carrara F. Viglione A. Lupori L. Lo Verde L. Benedetto A.. (2021). MEYE: web app for translational and real-time Pupillometry. eNeuro 8, ENEURO.0122–ENEU21.2021. doi: 10.1523/ENEURO.0122-21.2021, PMID: 34518364 PMC8489024

[ref130] McDougal D. H. Gamlin P. D. (2010). The influence of intrinsically-photosensitive retinal ganglion cells on the spectral sensitivity and response dynamics of the human pupillary light reflex. Vis. Res. 50, 72–87. doi: 10.1016/j.visres.2009.10.012, PMID: 19850061 PMC2795133

[ref131] McGrath E. R. Beiser A. S. DeCarli C. Plourde K. L. Vasan R. S. Greenberg S. M.. (2017). Blood pressure from mid- to late life and risk of incident dementia. Neurology 89, 2447–2454. doi: 10.1212/WNL.0000000000004741, PMID: 29117954 PMC5729797

[ref132] Mestanikova A. Ondrejka I. Mestanik M. Cesnekova D. Visnovcova Z. Bujnakova I.. (2017). Pupillary light reflex is altered in adolescent depression. Physiol. Res. 66, S277–S284. doi: 10.33549/physiolres.933683, PMID: 28937242

[ref133] Miller B. J. Sareddy S. Rosenquist P. B. McCall W. V. (2021). Pupillary light reflex markers of suicide risk in a trans-diagnostic sample. Schizophr. Res. 235, 1–2. doi: 10.1016/j.schres.2021.06.027, PMID: 34252639

[ref134] Mokdad A. H. Ford E. S. Bowman B. A. Dietz W. H. Vinicor F. Bales V. S.. (2003). Prevalence of obesity, diabetes, and obesity-related health risk factors, 2001. JAMA 289, 76–79. doi: 10.1001/jama.289.1.76, PMID: 12503980

[ref135] Nachtegaal J. Smit J. H. Smits C. Bezemer P. D. van Beek J. H. M. Festen J. M.. (2009). The association between hearing status and psychosocial health before the age of 70 years: results from an internet-based National Survey on hearing. Ear Hear. 30, 302–312. doi: 10.1097/AUD.0b013e31819c6e01, PMID: 19322094

[ref136] Noble K. A. (2010). Traumatic brain injury and increased intracranial pressure. J. Perianesthesia Nurs. 25, 242–250. doi: 10.1016/j.jopan.2010.05.008 20656261

[ref137] Oshorov A. V. Alexandrova E. V. Muradyan K. R. Sosnovskaya O. Y. Sokolova E. Y. Savin I. A. (2021). Pupillometry as a method for monitoring of pupillary light reflex in ICU patients. Vopr. Neirokhir. 85, 117–123. doi: 10.17116/neiro202185031117, PMID: 34156213

[ref138] Ott A. Slooter A. J. C. Hofman A. van Harskamp F. Witteman J. C. M. van Broeckhoven C.. (1998). Smoking and risk of dementia and Alzheimer’s disease in a population-based cohort study: the Rotterdam study. Lancet 351, 1840–1843. doi: 10.1016/s0140-6736(97)07541-7 9652667

[ref139] Paciência I. Rufo J. C. Silva D. Martins C. Mendes F. Rama T.. (2019). School environment associates with lung function and autonomic nervous system activity in children: a cross-sectional study. Sci. Rep. 9:15156. doi: 10.1038/s41598-019-51659-y, PMID: 31641175 PMC6805928

[ref140] Pan X. Luo Y. Roberts A. R. (2018). Secondhand smoke and Women’s cognitive function in China. Am. J. Epidemiol. 187, 911–918. doi: 10.1093/aje/kwx377, PMID: 29370335

[ref141] Park J. G. Moon C. T. Park D. S. Song S. W. (2015). Clinical utility of an automated Pupillometer in patients with acute brain lesion. J. Korean Neuro. Soc. 58, 363–367. doi: 10.3340/jkns.2015.58.4.363, PMID: 26587191 PMC4651998

[ref142] Park J. C. Moss H. E. McAnany J. J. (2016). The pupillary light reflex in idiopathic intracranial hypertension. Invest. Ophthalmol. Vis. Sci. 57, 23–29. doi: 10.1167/iovs.15-18181, PMID: 26746015 PMC4713014

[ref143] Perry D. C. Sturm V. E. Peterson M. J. Pieper C. F. Bullock T. Boeve B. F.. (2016). Association of traumatic brain injury with subsequent neurological and psychiatric disease: a meta-analysis. J. Neurosurg. 124, 511–526. doi: 10.3171/2015.2.JNS14503, PMID: 26315003 PMC4751029

[ref144] Piha S. J. Rönnemaa T. Koskenvuo M. (1994). Autonomic nervous system function in identical twins discordant for obesity. Int. J. Obes. Relat. Metab. Disord. 18, 547–550. PMID: 7951475

[ref145] Podcasy J. L. Epperson C. N. (2016). Considering sex and gender in Alzheimer disease and other dementias. Dialogues Clin. Neurosci. 18, 437–446. doi: 10.31887/DCNS.2016.18.4/cepperson, PMID: 28179815 PMC5286729

[ref146] Ponticorvo S. Manara R. Pfeuffer J. Cappiello A. Cuoco S. Pellecchia M. T.. (2019). Cortical pattern of reduced perfusion in hearing loss revealed by ASL-MRI. Hum. Brain Mapp. 40, 2475–2487. doi: 10.1002/hbm.24538, PMID: 30715769 PMC6865742

[ref147] Prettyman R. Bitsios P. Szabadi E. (1997). Altered pupillary size and darkness and light reflexes in Alzheimer’s disease. J. Neurol. Neurosurg. Psychiatry 62, 665–668. doi: 10.1136/jnnp.62.6.665, PMID: 9219763 PMC1074161

[ref148] Pronk M. Deeg D. J. H. Smits C. van Tilburg T. G. Kuik D. J. Festen J. M.. (2011). Prospective effects of hearing status on loneliness and depression in older persons: identification of subgroups. Int. J. Audiol. 50, 887–896. doi: 10.3109/14992027.2011.599871, PMID: 21929374

[ref149] Ralli M. Gilardi A. Stadio A. D. Severini C. Greco A. Vincentiis M.. (2019). Hearing loss and Alzheimer’s disease: a review. Int. Tinnitus J. 23, 79–85. doi: 10.5935/0946-5448.20190014 32009339

[ref150] Rehm J. Hasan O. S. M. Black S. E. Shield K. D. Schwarzinger M. (2019). Alcohol use and dementia: a systematic scoping review. Alzheimers Res. Ther. 11:1. doi: 10.1186/s13195-018-0453-0, PMID: 30611304 PMC6320619

[ref151] Reinvang I. Espeseth T. Westlye L. (2013). APOE-related biomarker profiles in non-pathological aging and early phases of Alzheimer's disease. Neurosci. Biobehav. Rev. 37, 1322–1335. doi: 10.1016/j.neubiorev.2013.05.006, PMID: 23701948

[ref152] Robles A. Touriño R. Gude F. Noya M. (1999). The tropicamide test in patients with dementia of Alzheimer type and frontotemporal dementia. Funct. Neurol. 14, 203–207. PMID: 10713893

[ref153] Rogers M. A. M. Langa K. M. (2010). Untreated poor vision: a contributing factor to late-life dementia. Am. J. Epidemiol. 171, 728–735. doi: 10.1093/aje/kwp453, PMID: 20150357 PMC2842219

[ref154] Romagnoli M. Stanzani Maserati M. de Matteis M. Capellari S. Carbonelli M. Amore G.. (2020). Chromatic pupillometry findings in Alzheimer’s disease. Front. Neurosci. 14:780. doi: 10.3389/fnins.2020.00780, PMID: 32848556 PMC7431959

[ref155] Romagnosi F. Bongiovanni F. Oddo M. (2020). Eyeing up the injured brain: automated pupillometry and optic nerve sheath diameter. Curr. Opin. Crit. Care 26, 115–121. doi: 10.1097/MCC.0000000000000710, PMID: 32068580

[ref156] Rubin L. S. (1980). Pupillometric studies of alcoholism. Int. J. Neurosci. 11, 301–308. doi: 10.3109/00207458009147594, PMID: 7451037

[ref157] Rusanen M. Kivipelto M. Quesenberry C. P. Zhou J. Whitmer R. A. (2011). Heavy smoking in midlife and long-term risk of Alzheimer disease and vascular dementia. Arch. Intern. Med. 171, 333–339. doi: 10.1001/archinternmed.2010.393, PMID: 20975015

[ref158] Sacks B. Smith S. (1989). People with Down’s syndrome can be distinguished on the basis of cholinergic dysfunction. J. Neurol. Neurosurg. Psychiatry 52, 1294–1295. doi: 10.1136/jnnp.52.11.1294, PMID: 2531786 PMC1031641

[ref159] Schor C. M. (1992). A dynamic model of cross-coupling between accommodation and convergence: simulations of step and frequency responses. Optom. Vis. Sci. 69, 258–269. doi: 10.1097/00006324-199204000-00002, PMID: 1565425

[ref160] Scinto L. F. Daffner K. R. Dressler D. Ransil B. I. Rentz D. Weintraub S.. (1994). A potential noninvasive neurobiological test for Alzheimer’s disease. Science (New York, N.Y.) 266, 1051–1054. doi: 10.1126/science.7973660, PMID: 7973660

[ref161] Segal O. Barak Lanciano S. Nussinovitch U. (2022). Association between body mass index and pupillary light reflex indices. Obesity Med. 32:100417. doi: 10.1016/j.obmed.2022.100417

[ref162] Selkoe D.J. (2012) Preventing Alzheimer’s disease. Available at: https://www.science.org/doi/full/10.1126/science.1228541 [Accessed February 20, 2022].

[ref163] Seshadri S. Wolf P. A. Beiser A. Au R. McNulty K. White R.. (1997). Lifetime risk of dementia and Alzheimer’s disease. The impact of mortality on risk estimates in the Framingham study. Neurology 49, 1498–1504. doi: 10.1212/wnl.49.6.1498, PMID: 9409336

[ref164] Sharma S. Baskaran M. Rukmini A. V. Nongpiur M. E. Htoon H. M. Cheng C. Y.. (2016). Factors influencing the pupillary light reflex in healthy individuals. Graefe’s Arch. Clin. Exp. Ophthalmol. 254, 1353–1359. doi: 10.1007/s00417-016-3311-4, PMID: 26968720

[ref165] Sheline Y. I. West T. Yarasheski K. Swarm R. Jasielec M. S. Fisher J. R.. (2014). An antidepressant decreases CSF Aβ production in healthy individuals and in transgenic AD mice. Sci. Transl. Med. 6:236re4. doi: 10.1126/scitranslmed.3008169, PMID: 24828079 PMC4269372

[ref166] Siegle G. J. Steinhauer S. R. Stenger V. A. Konecky R. Carter C. S. (2003). Use of concurrent pupil dilation assessment to inform interpretation and analysis of fMRI data. NeuroImage 20, 114–124. doi: 10.1016/s1053-8119(03)00298-2, PMID: 14527574

[ref167] Silva M. V. F. Loures C. M. G. Alves L. C. V. de Souza L. C. Borges K. B. G. Carvalho M. G. (2019). Alzheimer’s disease: risk factors and potentially protective measures. J. Biomed. Sci. 26:33. doi: 10.1186/s12929-019-0524-y, PMID: 31072403 PMC6507104

[ref168] Snyder P. J. Alber J. Alt C. Bain L. J. Bouma B. E. Bouwman F. H.. (2021). Retinal imaging in Alzheimer’s and neurodegenerative diseases. Alzheimers Dement. 17, 103–111. doi: 10.1002/alz.12179, PMID: 33090722 PMC8062064

[ref169] Sofi F. Valecchi D. Bacci D. Abbate R. Gensini G. F. Casini A.. (2011). Physical activity and risk of cognitive decline: a meta-analysis of prospective studies. J. Intern. Med. 269, 107–117. doi: 10.1111/j.1365-2796.2010.02281.x, PMID: 20831630

[ref170] Spitschan M. (2019). Photoreceptor inputs to pupil control. J. Vis. 19:5. doi: 10.1167/19.9.5, PMID: 31415056 PMC6699792

[ref171] Spitschan M. Jain S. Brainard D. H. Aguirre G. K. (2014). Opponent melanopsin and S-cone signals in the human pupillary light response. Proc. Natl. Acad. Sci. 111, 15568–15572. doi: 10.1073/pnas.1400942111, PMID: 25313040 PMC4217411

[ref172] Stein T. D. Alvarez V. E. McKee A. C. (2014). Chronic traumatic encephalopathy: a spectrum of neuropathological changes following repetitive brain trauma in athletes and military personnel. Alzheimers Res. Ther. 6:4. doi: 10.1186/alzrt234, PMID: 24423082 PMC3979082

[ref173] Steinhauer S. R. Siegle G. J. Condray R. Pless M. (2004). Sympathetic and parasympathetic innervation of pupillary dilation during sustained processing. Int. J. Psychophysiol. 52, 77–86. doi: 10.1016/j.ijpsycho.2003.12.005, PMID: 15003374

[ref174] Stergiou V. Fotiou D. Tsiptsios D. Haidich B. Nakou M. Giantselidis C.. (2009). Pupillometric findings in patients with Parkinson’s disease and cognitive disorder. Int. J. Psychophysiol. 72, 97–101. doi: 10.1016/j.ijpsycho.2008.10.010, PMID: 19047001

[ref175] Stern Y. (2012). Cognitive reserve in ageing and Alzheimer’s disease. Lancet Neurol. 11, 1006–1012. doi: 10.1016/S1474-4422(12)70191-6, PMID: 23079557 PMC3507991

[ref176] Strawbridge W. J. Wallhagen M. I. Shema S. J. Kaplan G. A. (2000). Negative consequences of hearing impairment in old age: a longitudinal analysis. The Gerontologist 40, 320–326. doi: 10.1093/geront/40.3.320, PMID: 10853526

[ref177] Suri S. Mackay C. E. Kelly M. E. Germuska M. Tunbridge E. M. Frisoni G. B.. (2015). Reduced cerebrovascular reactivity in young adults carrying the APOE ε4 allele. Alzheimers Dement. 11, 648–657.e1. doi: 10.1016/j.jalz.2014.05.1755, PMID: 25160043

[ref178] Tan E. T. Lambie D. G. Johnson R. H. Whiteside E. A. (1984). Parasympathetic denervation of the iris in alcoholics with vagal neuropathy. J. Neurol. Neurosurg. Psychiatry 47, 61–64. doi: 10.1136/jnnp.47.1.61, PMID: 6319614 PMC1027642

[ref179] Taylor W. R. Chen J. W. Meltzer H. Gennarelli T. A. Kelbch C. Knowlton S.. (2003). Quantitative pupillometry, a new technology: normative data and preliminary observations in patients with acute head injury: technical note. J. Neurosurg. 98, 205–213. doi: 10.3171/jns.2003.98.1.0205, PMID: 12546375

[ref180] Thompson H. S. (2005). Otto Lowenstein, Pioneer Pupillographer. J. Neuroophthalmol. 25, 44–49. doi: 10.1097/00041327-200503000-00012, PMID: 15756134

[ref181] Topiwala A. Allan C. L. Valkanova V. Zsoldos E. Filippini N. Sexton C.. (2017). Moderate alcohol consumption as risk factor for adverse brain outcomes and cognitive decline: longitudinal cohort study. BMJ 357:j2353. doi: 10.1136/bmj.j2353, PMID: 28588063 PMC5460586

[ref182] Truong J. Q. Ciuffreda K. J. (2016). Quantifying pupillary asymmetry through objective binocular pupillometry in the normal and mild traumatic brain injury (mTBI) populations. Brain Inj. 30, 1372–1377. doi: 10.1080/02699052.2016.1192220, PMID: 27712127

[ref183] Turana Y. Ranakusuma T. A. S. Purba J. S. Amir N. Ahmad S. A. Machfoed M. H.. (2014). Enhancing diagnostic accuracy of aMCI in the elderly: combination of olfactory test, pupillary response test, BDNF plasma level, and APOE genotype. IJAD 2014:e912586, 1–9. doi: 10.1155/2014/912586, PMID: 24639912 PMC3929508

[ref184] Tzekov R. Mullan M. (2014). Vision function abnormalities in Alzheimer disease. Surv. Ophthalmol. 59, 414–433. doi: 10.1016/j.survophthal.2013.10.002 24309127

[ref185] Valenti D. A. (2010). Alzheimer’s disease: visual system review. Optometry 81, 12–21. doi: 10.1016/j.optm.2009.04.101 20004873

[ref186] Valenzuela M. J. (2008). Brain reserve and the prevention of dementia. Curr. Opin. Psychiatry 21, 296–302. doi: 10.1097/YCO.0b013e3282f97b1f 18382231

[ref187] Valenzuela M. J. Sachdev P. (2006). Brain reserve and dementia: a systematic review. Psychol. Med. 36, 441–454. doi: 10.1017/S0033291705006264 16207391

[ref188] van der Flier W. M. Pijnenburg Y. A. L. Fox N. C. Scheltens P. (2011). Early-onset versus late-onset Alzheimer’s disease: the case of the missing APOE ɛ4 allele. Lancet Neurol. 10, 280–288. doi: 10.1016/S1474-4422(10)70306-9, PMID: 21185234

[ref189] van Stavern G. P. Bei L. Shui Y. B. Huecker J. Gordon M. (2019). Pupillary light reaction in preclinical Alzheimer’s disease subjects compared with normal ageing controls. Br. J. Ophthalmol. 103, 971–975. doi: 10.1136/bjophthalmol-2018-312425, PMID: 30206156

[ref190] Venkataraman A. Kalk N. Sewell G. Ritchie C. W. Lingford-Hughes A. (2017). Alcohol and Alzheimer’s disease-does alcohol dependence contribute to Beta-amyloid deposition, Neuroinflammation and neurodegeneration in Alzheimer’s disease? Alcohol Alcoholism 52, 151–158. doi: 10.1093/alcalc/agw092, PMID: 27915236

[ref191] Verma S. Hussain M. E. (2017). Obesity and diabetes: An update. Diabetes Metab. Syndr. Clin. Res. Rev. 11, 73–79. doi: 10.1016/j.dsx.2016.06.017 27353549

[ref192] Veronese N. Facchini S. Stubbs B. Luchini C. Solmi M. Manzato E.. (2017). Weight loss is associated with improvements in cognitive function among overweight and obese people: a systematic review and meta-analysis. Neurosci. Biobehav. Rev. 72, 87–94. doi: 10.1016/j.neubiorev.2016.11.017, PMID: 27890688

[ref193] Wang Z. Liu B. Zhu J. Wang D. Wang Y. (2019). Nicotine-mediated autophagy of vascular smooth muscle cell accelerates atherosclerosis via nAChRs/ROS/NF-κB signaling pathway. Atherosclerosis 284, 1–10. doi: 10.1016/j.atherosclerosis.2019.02.008, PMID: 30856513

[ref194] Wang L. Mao X. (2021). Role of retinal amyloid-β in neurodegenerative diseases: overlapping mechanisms and emerging clinical applications. Int. J. Mol. Sci. 22:2360. doi: 10.3390/ijms22052360 33653000 PMC7956232

[ref195] Wang Y. Zekveld A. A. Naylor G. Ohlenforst B. Jansma E. P. Lorens A.. (2016). Parasympathetic nervous system dysfunction, as identified by pupil light reflex, and its possible connection to hearing impairment. PLoS One 11:e0153566. doi: 10.1371/journal.pone.0153566, PMID: 27089436 PMC4835104

[ref196] Winn B. Whitaker D. Elliott D. B. Phillips N. J. (1994). Factors affecting light-adapted pupil size in normal human subjects. Invest. Ophthalmol. Vis. Sci. 35, 1132–1137. PMID: 8125724

[ref197] Wotton C. J. Goldacre M. J. (2014). Age at obesity and association with subsequent dementia: record linkage study. Postgrad. Med. J. 90, 547–551. doi: 10.1136/postgradmedj-2014-132571 25143590

[ref198] Xu T. Lan X. H. Guan Y. F. Zhang S. L. Wang X. Miao C. Y. (2015). Chronic nicotine treatment enhances vascular smooth muscle relaxation in rats. Acta Pharmacol. Sin. 36, 429–439. doi: 10.1038/aps.2015.5, PMID: 25832423 PMC4387309

[ref199] Yaffe K. Lwi S. J. Hoang T. D. Xia F. Barnes D. E. Maguen S.. (2019). Military-related risk factors in female veterans and risk of dementia. Neurology 92, e205–e211. doi: 10.1212/WNL.0000000000006778, PMID: 30541865 PMC6340384

[ref200] Yang Y. Yu Y. Yao K. (2006). Pupillary dysfunction in type 2 diabetes mellitus to refine the early diagnosis of diabetic autonomic neuropathy. Neuro-Ophthalmology 30, 17–21. doi: 10.1080/01658100600599527

[ref201] Yoshiyama S. Chen Z. Okagaki T. Kohama K. Nasu-Kawaharada R. Izumi T.. (2014). Nicotine exposure alters human vascular smooth muscle cell phenotype from a contractile to a synthetic type. Atherosclerosis 237, 464–470. doi: 10.1016/j.atherosclerosis.2014.10.019, PMID: 25463075

[ref202] Zandi B. Lode M. Herzog A. Sakas G. Khanh T. Q. (2021). PupilEXT: flexible open-source platform for high-resolution pupillometry in vision research. Front. Neurosci. 15:676220. doi: 10.3389/fnins.2021.676220, PMID: 34220432 PMC8249868

[ref203] Zanier E. R. Bertani I. Sammali E. Pischiutta F. Chiaravalloti M. A. Vegliante G.. (2018). Induction of a transmissible tau pathology by traumatic brain injury. Brain J. Neurol. 141, 2685–2699. doi: 10.1093/brain/awy193, PMID: 30084913 PMC6113646

[ref204] Zekveld A. A. Kramer S. E. Festen J. M. (2011). Cognitive load during speech perception in noise: the influence of age, hearing loss, and cognition on the pupil response. Ear Hear. 32, 498–510. doi: 10.1097/AUD.0b013e31820512bb, PMID: 21233711

[ref205] Zekveld A. A. van Scheepen J. A. M. Versfeld N. J. Kramer S. E. van Steenbergen H. (2020). The influence of hearing loss on cognitive control in an auditory conflict task: behavioral and Pupillometry findings. J. Speech Lang. Hear. Res. 63, 2483–2492. doi: 10.1044/2020_JSLHR-20-00107 32610026

